# Rhizosphere-associated soil microbiome variability in *Verticillium* wilt-affected *Cotinus coggygria*

**DOI:** 10.3389/fmicb.2023.1279096

**Published:** 2024-01-05

**Authors:** Juan Zhao, Yanli Cheng, Nan Jiang, Guanghang Qiao, Wentao Qin

**Affiliations:** ^1^Institute of Plant Protection, Beijing Academy of Agriculture and Forestry Sciences, Beijing, China; ^2^Beijing Key Laboratory of Environment Friendly Management on Fruit Diseases and Pests in North China, Beijing Academy of Agriculture and Forestry Sciences, Beijing, China; ^3^College of Life Sciences, Yangtze University, Jingzhou, Hubei, China; ^4^College of Horticulture and Plant Protection, Inner Mongolia Agricultural University, Hohhot, Inner Mongolia, China

**Keywords:** *Cotinus coggygria*, *Verticillium* wilt, soil microbiome, rhizosphere, plant health, Illumina sequencing

## Abstract

**Introduction:**

*Verticillium* wilt is the most devastating soil-borne disease affecting *Cotinus coggygria* in the progress of urban landscape construction in China.

**Methods:**

To assess the variability of the rhizosphere-associated soil microbiome in response to *Verticillium* wilt occurrence, we investigated the microbial diversity, taxonomic composition, biomarker species, and co-occurrence network of the rhizosphere-associated soil in *Verticillium* wilt*-*affected *C. coggygria* using Illumina sequencing.

**Results:**

The alpha diversity indices of the rhizosphere bacteria in *Verticillium* wilt-affected plants showed no significant variability compared with those in healthy plants, except for a moderate increase in the Shannon and Invsimpson indices, while the fungal alpha diversity indices were significantly decreased. The abundance of certain dominant or crucial microbial taxa, such as *Arthrobacter*, *Bacillus*, *Streptomyces*, and *Trichoderma*, displayed significant variations among different soil samples. The bacterial and fungal community structures exhibited distinct variability, as evidenced by the Bray–Curtis dissimilarity matrices. Co-occurrence networks unveiled intricate interactions within the microbial community of *Verticillium* wilt-affected *C. coggygria*, with greater edge numbers and higher network density. The phenomenon was more evident in the fungal community, showing increased positive interaction, which may be associated with the aggravation of *Verticillium* wilt with the aid of *Fusarium*. The proportions of bacteria involved in membrane transport and second metabolite biosynthesis functions were significantly enriched in the diseased rhizosphere soil samples.

**Discussion:**

These findings suggested that healthy *C. coggygria* harbored an obviously higher abundance of beneficial microbial consortia, such as *Bacillus,* while *Verticillium* wilt-affected plants may recruit antagonistic members such as *Streptomyces* in response to *Verticillium dahliae* infection. This study provides a theoretical basis for understanding the soil micro-ecological mechanism of *Verticillium* wilt occurrence, which may be helpful in the prevention and control of the disease in *C. coggygria* from the microbiome perspective.

## Introduction

*Cotinus coggygria* Scop., also known as the smoke tree, is one of the most important landscape trees worldwide with ornamental, medicinal, and economic values (Alexandra-Gabriela et al., 2023). It is widely planted as a well-known red leaf plant in China, especially in Fragrant Hill and other famous scenic spots in the Beijing municipality. *Verticillium* wilt caused by *Verticillium dahliae* Kleb. is considered the most devastating disease in many regions where *C. coggygria* is cultivated (Lei, 1993). The main manifestations of *Verticillium* wilt in *C. coggygria* is vascular bundle blockage, early deciduous growth, and stunted growth, which not only destroy the red leaf landscape but also cause massive tree mortality (Zhou et al., 2019). As a typical soil-borne pathogen, *V. dahliae* is characterized by strong temperature adaptation and a wide host range, and can harm more than 200 species of plants (Tian et al., 2016). Owing to *V. dahliae’s* long latency and it being prone to develop drug resistance, conventional prevention and control measures were inefficient or difficult to implement on *Verticillium* wilt (Xiong et al., 2014). With the continuous expansion of forest areas and the reduction of tree species, the *Verticillium* wilt disease is becoming more serious, which negatively influences the sustainable cultivation of *C. coggygria* (Tian et al., 2016).

Plants select and shape the rhizosphere-associated soil microbiome, stimulating or repressing certain members of the indigenous microbial communities, which usually act as the first defense line against soil-borne pathogens through a range of mechanisms, including direct pathogen inhibition, space and nutrition competition, and antibiotic production (Berg et al., 2017; Park et al., 2023). Numerous previous studies have demonstrated the variability of plant rhizosphere-associated soil microbiome, such as olives with *Verticillium* wilt occurrence (Fernández-González et al., 2020), ficus trees affected by brown root rot (Liu et al., 2022), and poplars under different genotypes and soil conditions (Karliński et al., 2020). Rhizosphere-associated microbiota, which closely interact with plant roots, are important for plant health maintenance and disease resistance induction (Wei et al., 2019). It becomes a central issue for present research in both forestry and ecology fields to unravel how soil microbial communities change in response to pathogen infection (Liao et al., 2022).

Understanding the association between disease occurrence and rhizosphere-associated microbiome may provide a basis for manipulating soil microbiome to promote plant health (Wei et al., 2021). Zhou et al. (2019) have reported the dynamic changes in bacterial diversity and community structure of *C. coggygria* plants from different years, geographic areas, and landscape configurations. However, studies were rarely investigated to understand the nature of the rhizosphere-associated soil microbiome in *C. coggygria* in response to *Verticillium* wilt occurrence. Therefore, we aimed to characterize the microbial diversity, taxonomic composition, biomarker species, and co-occurrence network of the rhizosphere and non-rhizosphere soil microbiome of *C. coggygria* under *Verticillium* wilt-affected and healthy conditions in the hope of providing a theoretical foundation for the scientific management of *Verticillium* wilt.

## Materials and methods

### Site description and sample collection

The soil samples were collected from the northern slope of Fragrant Hill (39°99′61′′N and 116°19′50′′E) in the Haidian district of Beijing, China, in August 2021. Fragrant Hills is the best-known *Cotinus coggygria* planting region in China, which is also a typical region of *Verticillium* wilt occurrence. This region has a sub-humid, warm temperate continental monsoon climate with an annual precipitation of approximately 600 mm. The typical symptoms of *Verticillium* wilt were foliar chlorosis and necrosis, stunting, vascular discoloration, die-back of individual branches, and death of the entire tree (Wang et al., 2013). For each plant, the *Verticillium* wilt disease rating was assessed on a scale of 0 to 4, based on the proportion of yellowing or wilting leaves on the entire plant (Wang et al., 2013). In this study, the *C. coggygria* plants that showed distinct *Verticillium* wilt symptoms on more than two-thirds of the stems based on disease rating and were positive for pathogen isolation were classified as diseased plants, while the plants with no wilting symptoms and were negative for pathogen isolation were considered healthy plants.

Typical diseased and healthy plants of *C. coggygria* with similar ages and sizes in the same *C. coggygria* forest district were selected to obtain their rhizosphere-associated soil samples (Edwards et al., 2015; Zhou et al., 2021), with three replications each group and five plants each replication. After carefully digging out the fibrous root systems of plants, the loose soil particles and large clods removed from the roots were collected as non-rhizosphere soil. The soils tightly adhered to the surface of the roots were washed with phosphate-buffered saline, and then the rhizosphere soil was reserved after the centrifugation of the soil slurry (Zhou et al., 2021). In addition, the belowground soil in the *C. coggygria* forest district, approximately 80–100 cm horizontally away from plant roots, was sampled as bulk soil. The soil samples from five plants in each replication were combined as a single composite sample. Therefore, five soil sample groups, including the rhizosphere soil from healthy and *Verticillium* wilt-affected plants (RHS and RPS), the non-rhizosphere soil from healthy and affected plants (NRHS and NRPS), as well as the bulk soil in the *C. coggygria* forest (LS), were obtained with three replications. All samples were placed in aseptic bags, transported back to the lab, and treated using a 2 mm sieve. Each sample was divided into two parts: one part was air-dried to measure the physicochemical properties, and the other part was stored at −80°C for DNA extraction.

### Measurement of soil physicochemical properties

The physicochemical properties of the *C. coggygria* soil samples, such as soil organic carbon, total nitrogen, total phosphorus, total potassium, ammonium nitrogen, nitrate nitrogen, available phosphorus, available potassium, and pH, were tested following the methods of Bao (2000). Total organic carbon (TOC) was measured by the K_2_Cr_2_O_7_-H_2_SO_4_ oxidation method in an automated TOC analyzer (TOC-VCPH; Shimadzu, Shimane-ken, Japan) following the manufacturer’s instructions. Total nitrogen (TN) was determined by the Kjeldahl procedure with a carbon–hydrogen–nitrogen (CHN) elemental analyzer (Perkin Elmer; Boston, MA, USA). Total phosphorus (TP) was measured by the molybdenum antimony colorimetric method with an ultraviolet–visible spectrophotometer (UV-2550; Shimadzu, Kyoto, Japan). Total potassium (TK) was measured by the NaOH fusion flame emission technique with a flame photometer (FP6400; Drawell, Shanghai, China). Soil-available phosphorus (AVP) was extracted with 0.5 mol·L^−1^ of NaHCO_3_ and determined using the molybdenum blue method. Soil-available potassium (AVK) was extracted with 1 mol·L^−1^ of NH_4_Ac and determined using the flame emission technique. The soil ammonium nitrogen (NH_4_^+^-N) and nitrate nitrogen (NO_3_^−^-N) concentrations were determined by an automated flow injection analyzer (FS3100; Xylem, Washington, United States) after the extraction of the soil samples with 2 mol·L^−1^ of KCl. Soil pH was determined with a pH meter (ST2100; OHRUS, Jiangsu, China) with a soil-to-water ratio of 1:2.5 (Marcos et al., 2019).

### DNA extraction, library preparation, and sequencing

Total genomic DNA was extracted using the Power Soil DNA Isolation Kit (Mo Bio Laboratories, Inc., USA) according to the standard protocols. The concentration and purity of the extracted DNA were determined using a NanoDrop 2000 UV–Vis spectrophotometer (Thermo Fisher Scientific, Wilmington, United States). The DNA quality was checked by 1% agarose gel electrophoresis. The V3–V4 variable region of the bacterial 16S rRNA gene was amplified using the primers 338F and 806R (Zhou et al., 2017). The fungal rDNA-ITS sequence was amplified using the primers ITS1F and ITS2R (Adams et al., 2013; Mcguire et al., 2013). High-throughput sequencing was performed using the Illumina MiSeq sequencing platform at Majorbio Bio-Pharm Technology Co., Ltd. (Shanghai, China).

### Data processing and sequence analysis

Raw sequence files were analyzed and quality-filtered with the Quantitative Insights Into Microbial Ecology (QIIME 1.9.1 software, http://qiime.org/install/index.html). Sequences were screened at 97% similarity using UPRASE (version 7.0.1090) with the help of an agglomerative clustering algorithm (Edgar, 2013). The taxonomic identities of the bacteria and fungi were determined using the SILVA database^1^ and the Unite database,^2^ respectively. All sequences were deposited in the NCBI Sequence Read Archive with the submission accession number SAMN35564492-SAMN35564506 under BioProject ID PRJNA 979017 for the bacterial community and SAMN35564971-SAMN35564985 under BioProject ID PRJNA 979102 for the fungal community. Functional prediction of the microbial community was performed using PICRUSt2 based on the Kyoto Encyclopedia of Genes and Genomes (KEGG) database (Douglas et al., 2019), Bugbase, and FUNGuild (Nguyen et al., 2016) on the cloud platform of Majorbio Bio-Pharm Technology Co., Ltd., Shanghai, China.^3^

### Statistical analyses

All the data were displayed as the mean ± standard deviation of three replications. The significance of the differences (*p* < 0.05) was evaluated by the least significant difference (LSD) test after a one-way analysis of variance (ANOVA) using the SPSS 17.0 software. Alpha diversity analysis of the microbial community was performed by Mothur software version 1.30.2 (Schloss et al., 2009). The beta diversity was calculated based on Bray–Curtis distance metrices and visualized by principal coordinate analysis (PCoA) in QIIME (Caporaso et al., 2010). Linear discriminant analysis was conducted by LEfSe software^4^ to identify the microbial biomarkers at different taxonomic levels (Segata et al., 2011). The strong (ρ > 0.8 or < −0.8) and significant (adjusted *p*-value < 0.01) correlations were retained for use in co-occurrence network construction at the genus level. Network visualization and topological analysis were performed using Gephi v0.9.2.

## Results

### Sequencing data analyses

Through high-throughput sequencing analyses of 16S rRNA and the ITS region, a total of 6,133 operational taxonomic units (OTUs) were obtained for the bacteria, which were further assigned into 39 phyla, 127 classes, 300 orders, 479 families, and 876 genera, including some unclassified groups, whereas 2,579 OTUs attributed to 14 phyla, 51 classes, 125 orders, 282 families, and 604 genera were obtained for the fungi. According to the Venn diagrams, a total of 2,212 bacterial OTUs were shared across all the soil samples, while 113~246 unique bacterial OTUs were exclusively detected in each sample. In addition, a total of 396 fungal OTUs were shared, and 72~286 fungal OTUs were uniquely detected (Supplementary Figure S1).

### Alpha diversity variation in different soil samples

**Figure 1:**
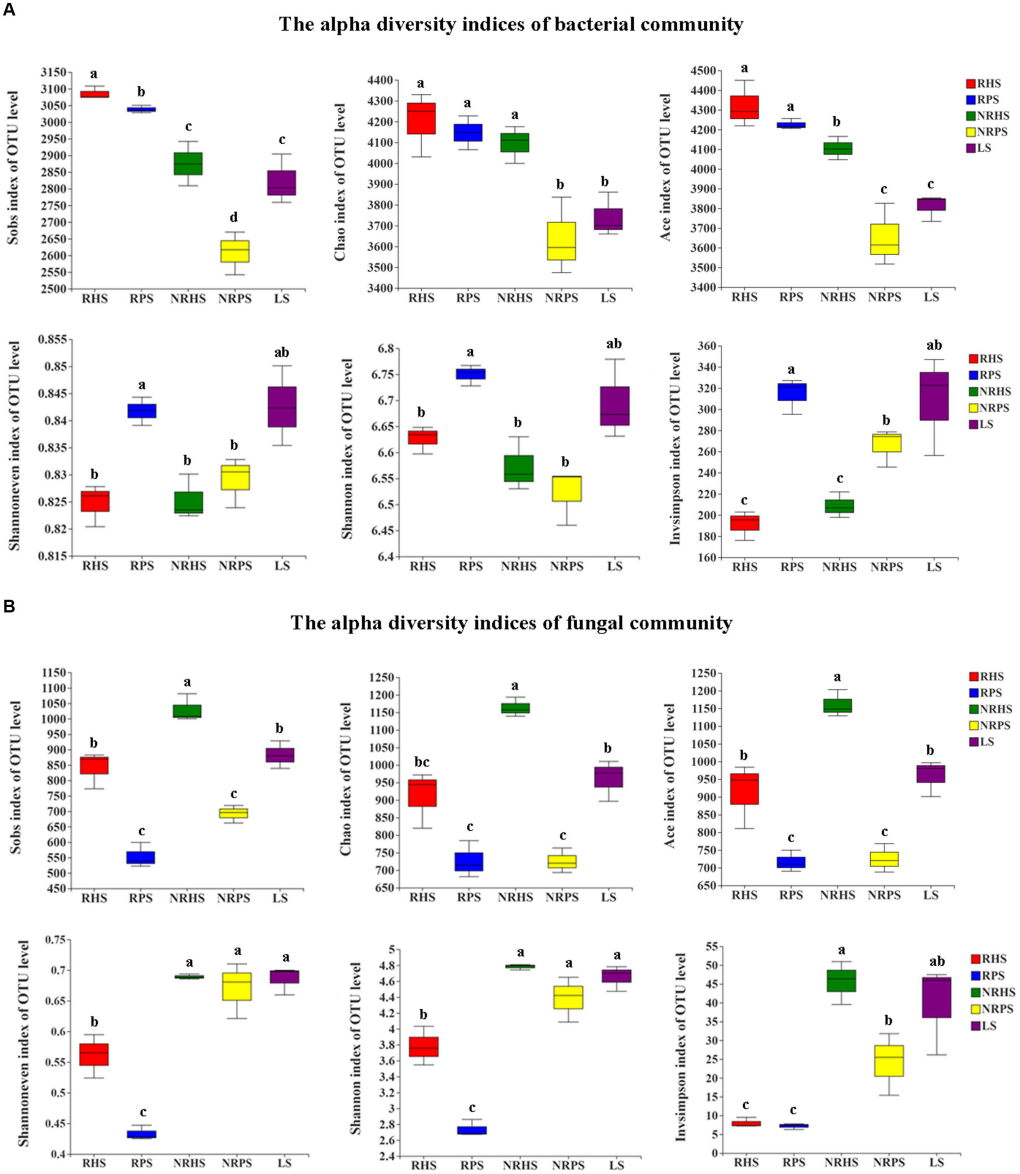
Boxplots of the alpha diversity indices of bacterial **(A)** and fungal **(B)** OTUs in the *Cotinus coggygria* soil microbiome under different conditions. Different lowercase letters denoted significant differences at *p*-values < 0.05 levels among different soil samples. RHS, rhizosphere soil from healthy plants; RPS, rhizosphere soil from *Verticillium* wilt-affected plants; NRHS, non-rhizosphere soil from healthy plants; NRPS, non-rhizosphere soil from *Verticillium* wilt-affected plants; LS, bulk soil in the *C. coggygria* forest.

Alpha diversity of the bacterial OTUs indicated that significant differences in richness indices were detected between the NRHS and NRPS soil samples; the Sobs, Chao, and Ace indices decreased significantly in the non-rhizosphere soil of *Verticillium* wilt-affected *C. coggygria*. For evenness and diversity estimates, we found a clear separation between the RHS and RPS soil samples (*p* < 0.05), with higher values observed for the RPS sample (Figure 1A). In comparison to the alpha diversity indices of the fungal community, the Sobs, Chao, and Ace indices were significantly decreased in *Verticillium* wilt-affected *C. coggygria* soil samples (*p* < 0.05). The Shannoneven and Shannon indices of the RPS sample, as well as the Invsimpson index of the NRPS sample, were also obviously reduced (Figure 1B).

### Community composition variation in different soil samples

Considering the relative abundances of the bacterial taxa, Actinobacteria (30.5%–46.5%) was the major phylum, followed by Proteobacteria (16.9%–25.3%), Acidobacteria (7.1%–21.6%), Chloroflexi (6.2%–12.5%), Firmicutes (1.1%–8.9%), and Gemmatimonadota (2.2%–3.9%), accounting for more than 87% of the total bacterial abundance (Supplementary Table S1). The primary bacteria at the class level consisted of Actinobacteria, Alphaproteobacteria, Thermoleophilia, Vicinamibacteria, and Gammaproteobacteria (Supplementary Figure S2). The predominant fungal phyla were Ascomycota (47.6%–82.6%), followed by Basidiomycota (9.9%–50.5%) and Mortierellomycota (0.5%–10.2%), accounting for more than 92% of the total abundance (Supplementary Table S1). It was clear that the abundance difference in some fungal taxa explained the obvious variation in the community composition of *Verticillium* wilt-affected soil samples (Supplementary Figure S2).

**Figure 2:**
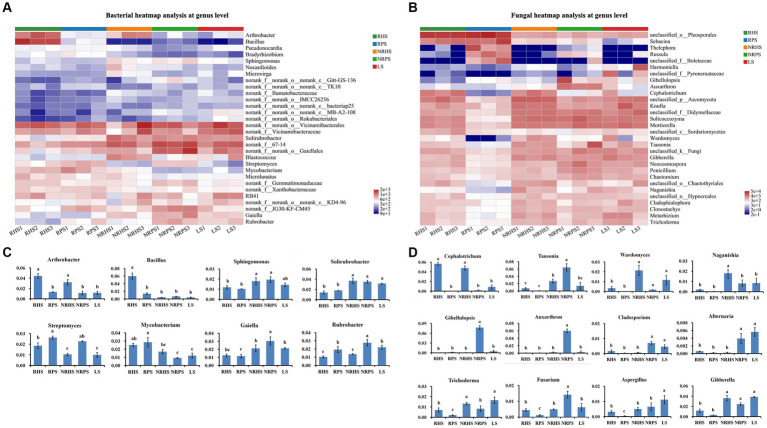
Relative abundances of the dominant bacterial and fungal taxa in the *Cotinus coggygria* soil microbiome under different conditions. **(A)** Heatmap representing the relative abundance of the top 30 bacterial taxa at the genus level, **(B)** average relative abundance of bacterial genera with significant differences among different soil samples, **(C)** heatmap representing the relative abundance of the top 30 fungal taxa at the genus level, and **(D)** average relative abundance of fungal genera with crucial features or significant differences among the soil samples. The x-axis represented samples, and the y-axis represented relative abundance. The error bar represented the standard deviation of mean values. Different lowercase letters showed significant differences in abundance among different soil samples based on the LSD test (*p* < 0.05). RHS, rhizosphere soil from healthy plants; RPS, rhizosphere soil from *Verticillium* wilt-affected plants; NRHS, non-rhizosphere soil from healthy plants; NRPS, non-rhizosphere soil from *Verticillium* wilt-affected plants; LS, bulk soil in the *C. coggygria* forest.

Heatmap analysis of the top 30 bacterial taxa at the genus level (Figure 2A) showed that the bacterial genera mainly consisted of *Arthrobacter*, *Bacillus*, *Streptomyces*, and *Rubrobacter*, whose abundance significantly differed between rhizosphere soil samples of *Verticillium* wilt-affected and healthy *C. coggygria*, despite some similar soil physicochemical characteristics such as total nitrogen, total phosphorus, total potassium, available phosphorus, nitrate nitrogen, and pH (Supplementary Figure S3). As shown in Figure 2B, the genus *Bacillus* was significantly enriched in the RHS soil sample, while *Streptomyces* was obviously enriched in the RPS and NRPS samples.

**Figure 3:**
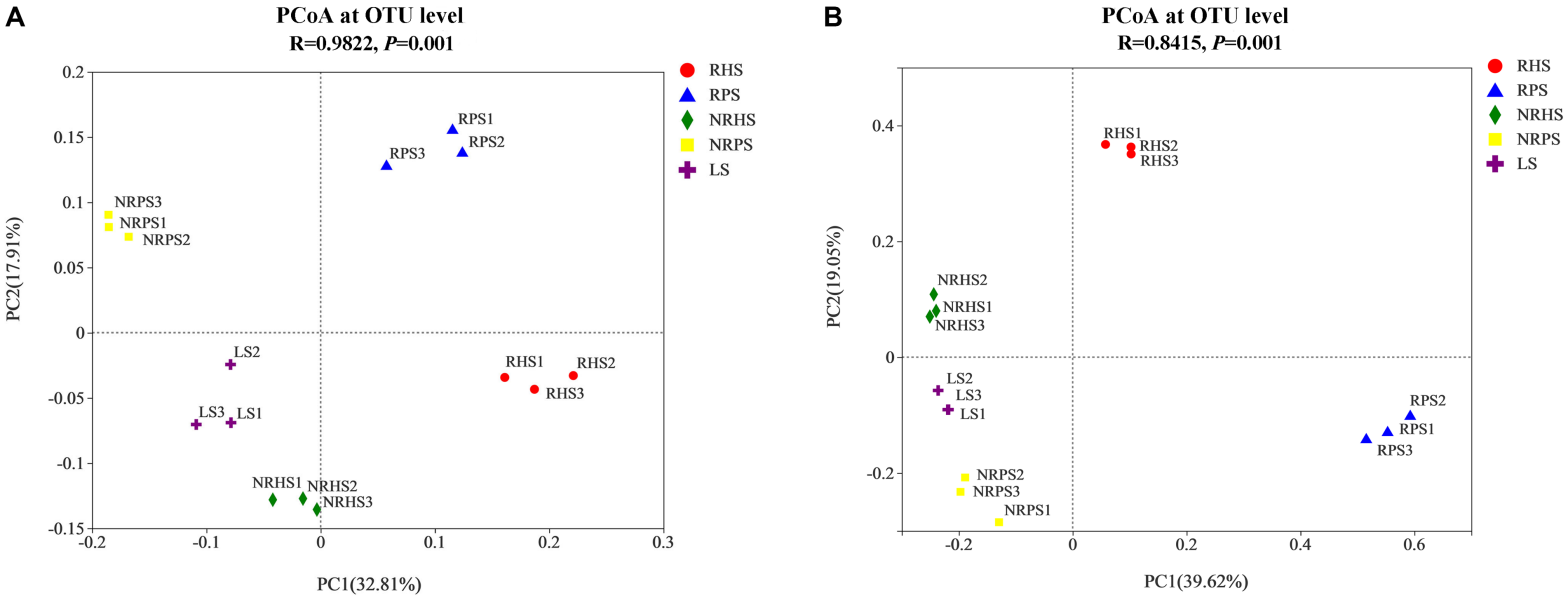
Principal coordinate analysis (PCoA) of bacterial **(A)** and fungal **(B)** communities of the *Cotinus coggygria* soil microbiome under different conditions. The PCoA plots were based on the Bray–Curtis dissimilarity of bacterial and fungal communities at the OTU levels according to the ANOSIM test. RHS, rhizosphere soil from healthy plants; RPS, rhizosphere soil from *Verticillium* wilt-affected plants; NRHS, non-rhizosphere soil from healthy plants; NRPS, non-rhizosphere soil from *Verticillium* wilt-affected plants; LS, bulk soil in the *C. coggygria* forest.

The relative abundances of genera *Cephalotrichum* and *Wardomyces* were lower in the soil samples of *Verticillium* wilt-affected *C. coggygria*, while the genus *Russula* obviously increased in abundance (Figure 2C). We also compared some well-known fungal genera and found that *Trichoderma* was significantly increased in both rhizosphere and non-rhizosphere soils of the healthy plants (Figure 2D). The variations in some crucial microbial species were further compared among soil samples based on a 95% confidence interval and Welch’s *t*-test (Supplementary Figures S4, S5). Principal coordinate analysis (PCoA) indicated that the bacterial community structure was distinctly separated based on soil compartments and plant health status, with the PC1 axis showing 32.81% variation and the PC2 axis explaining 17.91% (Figure 3A). Soil compartments and plant health status also explained 39.62% and 19.05% of the variability in fungal community composition, respectively (Figure 3B).

### Microbial biomarkers variation in different soil samples

**Figure 4:**
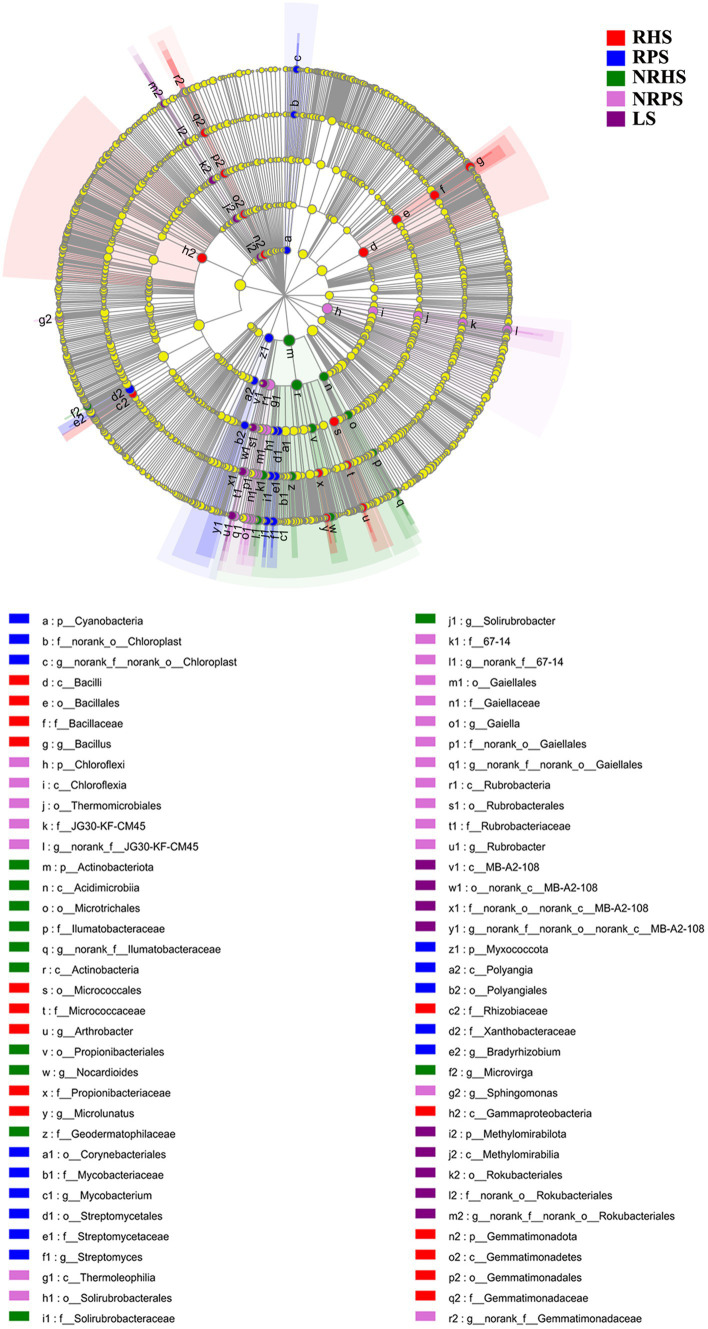
Linear discriminant analysis effect size (LEfSe) of bacterial taxa in the *Cotinus coggygria* soil microbiome under different conditions. The non-parametric factorial Kruskal–Wallis test with *p* < 0.05 and logarithmic LDA score > 3.5 was used to identify the significantly enriched bacterial taxa (using the all-against-all comparisons parameter). Different colors depicted different soil samples, while circles from inside to outside showed phylogenetic levels from phylum to genera. The sizes of the circles were proportional to the mean relative abundance of each taxon. The yellow circles represented the absence of significantly different taxa. Genera with a relative abundance of less than 0.1% were not included.

**Figure 5:**
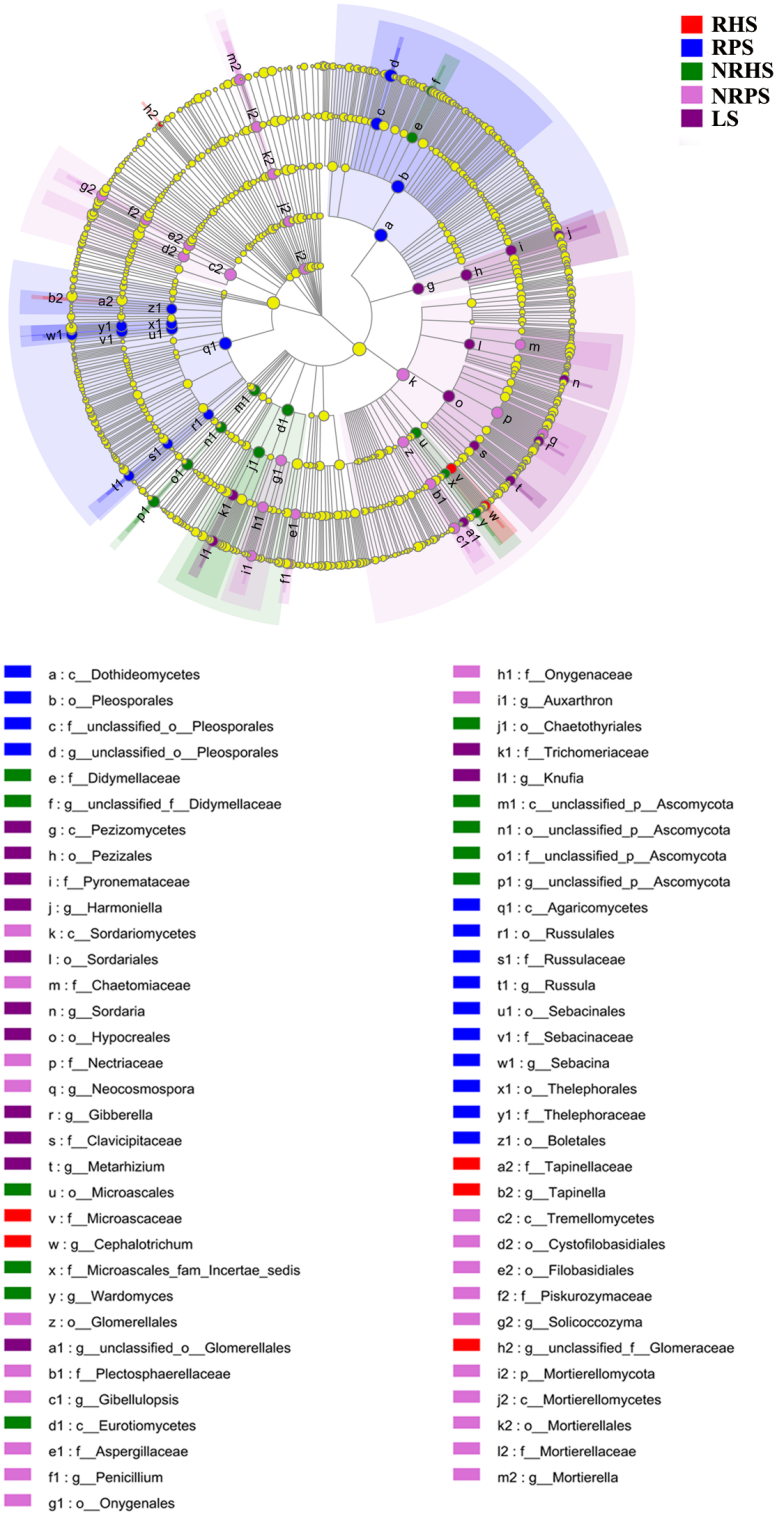
Linear discriminant analysis effect size (LEfSe) of fungal taxa in the *Cotinus coggygria* soil microbiome under different conditions. The non-parametric factorial Kruskal–Wallis test with *p* < 0.05 and logarithmic LDA score > 3.5 was used to identify the significantly enriched fungal taxa (using the all-against-all comparisons parameter). The notes were the same as Figure 4.

Linear discriminant analysis effect size (LEfSe) was used to scan the biomarkers in the soil microbiome of *C. coggygria* from different soil compartments and plant health status. At the phylum level, Gemmatimonadota was significantly enriched in the RHS sample, while Cyanobacteria and Myxococcota were enriched in the RPS sample. The phylum Actinobacteriota, Chloroflexia, and Methylomirabilota represented the biomarkers of the NRHS, NRPS, and LS samples, respectively. At the genus level, *Bacillus*, *Arthrobacter*, and *Microlunatus* were significantly enriched in the RHS sample, while *Mycobacterium*, *Streptomyces*, and *Bradyrhizobium* were more abundant in the RPS sample (Figure 4). LEfSe analysis based on the fungal genera also displayed some unique biomarkers in different soil samples (Figure 5).

The histogram of the LDA effect value indicated that *Bacillus* (from class to genus), *Micrococcaceae* (from order to family), the phylum Firmicutes, and the genus *Arthrobacter* were the most decisive bacterial members in the RHS soil sample, while *Mycobacterium* (from family to genus) and *Streptomyces* (from order to genus) were obviously the biomarkers in the RPS sample. In addition, the relative abundance of *Actinobacteria* (from phylum to class) and *Thermomicrobiales* (from class to order) were the most apparent taxa in NRHS and NRPS soil samples, respectively (Supplementary Figure S6). On the other hand, the genera *Cephalotrichum* and *Tapinella* were the most influential members, leading to a distinct fungal community in RHS, while the genera *Sebacina* and *Russula* were the biomarkers in RPS (Supplementary Figure S7).

### Interaction network variation in different soil samples

To gain a deeper insight into the interaction variability of the soil microbiome in *Verticillium* wilt-affected *Cotinus coggygria*, the microbial communities in the rhizosphere and non-rhizosphere soil samples were merged. Results showed that the microbial networks of *Verticillium* wilt-affected plants differed profoundly from the healthy networks, with higher average degrees, degree centrality, closeness centrality, and network density (Supplementary Table S2). More positive interactions occurred in the healthy bacterial network (BHS, 68.6%, 450 edges) than in the diseased soil samples (BPS, 52.9%, 546 edges). In the fungal network, the positive interactions of the diseased network (FPS, 84.6%, 981 edges) were significantly increased compared to the healthy samples (FHS, 62.5%, 450 edges). The ratio of positive to negative interactions (P/N ratio) was decreased in the bacterial network of *Verticillium* wilt-affected plants, while those of the fungal network were obviously increased from 1.7 to 5.5 (Supplementary Figure S8). All these suggested that the disease status strengthened the microbial nodes and their associations in the microbial community, especially the fungal co-occurrence network.

**Figure 6:**
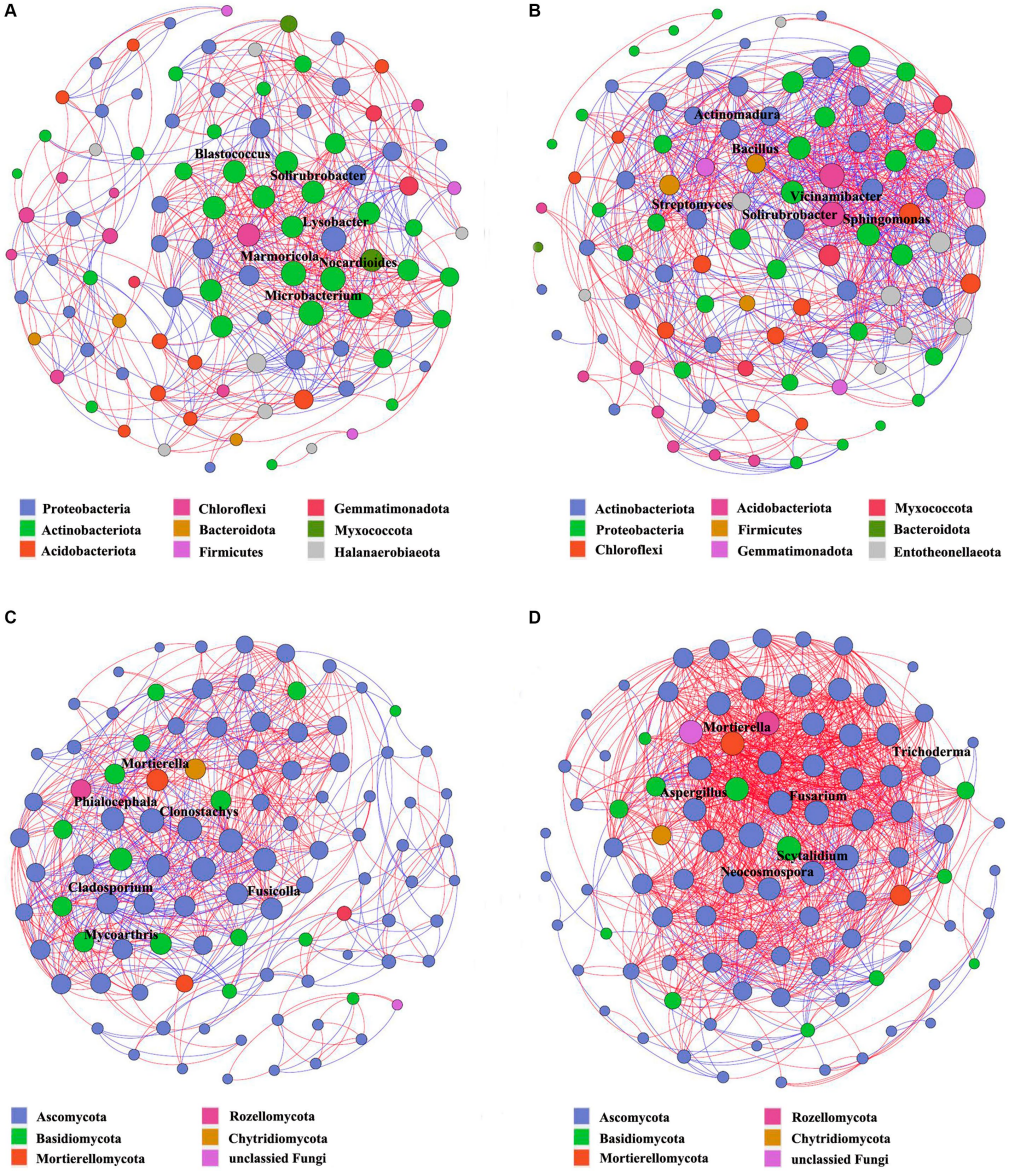
Co-occurrence networks constructed based on bacterial and fungal communities in the *Cotinus coggygria* soil microbiome under different conditions. **(A)** Bacterial networks of soil samples from healthy plants, BHS, **(B)** bacterial networks of soil samples from *Verticillium* wilt-affected plants, BPS, **(C)** fungal networks of soil samples from healthy plants, FHS, and **(D)** fungal networks of soil samples from *Verticillium* wilt-affected plants, FPS. The nodes were colored based on phyla, and each node size was proportional to the degree of centrality. A connection denotes a strong (Spearman’s *ρ* > 0.8 or < −0.8, red or blue edges) and significant (*p* < 0.05) correlation. Potential keystone taxa were shown with bold genera names.

We also found that the nodes representing genera *Blastococcus*, *Solirubrobacter*, *Marmoricola*, *Nocardioides*, and *Microbacterium* belonging to Actinobacteria and genus *Lysobacter* from Proteobacteria were closely related to other bacterial genera with higher degrees in the healthy network (Supplementary Table S3; Figure 6A). The genera *Streptomyces*, *Solirubrobacter*, and *Actinomadura* belonging to *Actinobacteriota*, as well as *Vicinamibacter* (Acidobacteriota), *Sphingomonas* (Proteobacteria), and *Bacillus* (Firmicutes), occupied critical positions in the diseased network and had important impacts on other bacterial groups (Supplementary Table S4; Figure 6B). In the soil fungal co-occurrence network of healthy plants (Figure 6C), the genera *Phialocephala*, *Fusicolla*, *Mycoarthris*, *Clonostachys,* and *Cladophialophora* belonging to Ascomycota, as well as the genus *Mortierella* belonging to Basidiomycota, displayed the highest degree, degree centrality, or betweenness centrality (Supplementary Table S5). The nodes representing the genera *Fusarium*, *Neocosmospora*, *Mortierella*, *Trichoderma*, *Aspergillus*, and *Scytalidium* were closely related to other genera, and their degrees of connection were the highest in the diseased network (Supplementary Table S6). Among which the genus *Fusarium* had 29 positively related genera, such as *Metarhizium*, *Penicillium*, *Wardomyces*, *Mortierella*, and *Cephalotrichum* (Figure 6D).

### Microbial function variation in different soil samples

**Figure 7:**
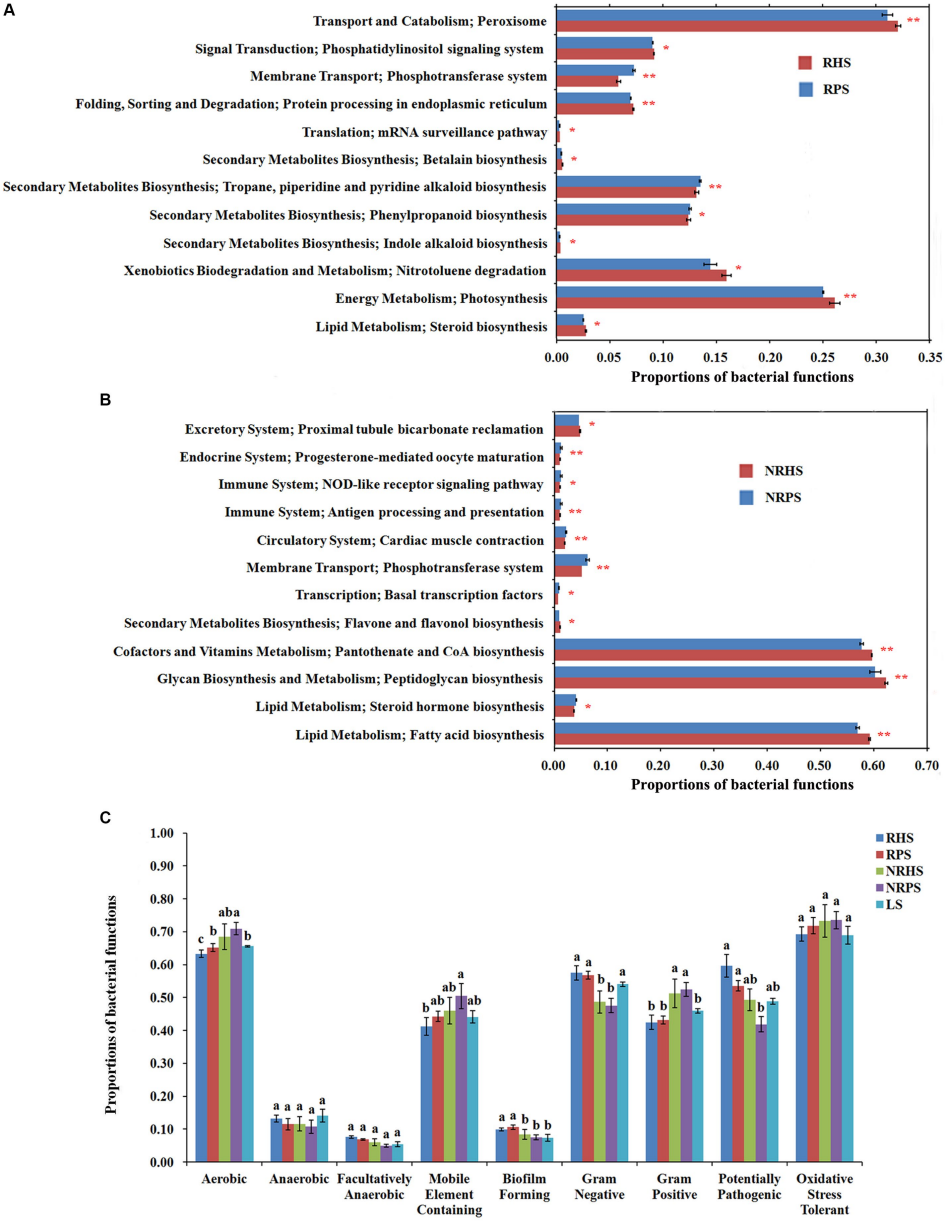
Proportions of bacterial functions in the *Cotinus coggygria* soil microbiome under different conditions based on KEGG and Bugbase databases. **(A,B)** KEGG ortholog function for bacteria in the rhizosphere **(A)** and non-rhizosphere **(B)** soil samples, **(C)** bacteria function profiles in different soil samples based on Bugbase prediction. RHS, rhizosphere soil from healthy plants; RPS, rhizosphere soil from *Verticillium* wilt-affected plants; NRHS, non-rhizosphere soil from healthy plants; NRPS, non-rhizosphere soil from *Verticillium* wilt-affected plants; LS, bulk soil in *C. coggygria* forest.

The distribution of bacterial function was significantly different in the soil microbiome of *Verticillium* wilt-affected *Cotinus coggygria*. The functions connected with transport and catabolism, folding, sorting, and degradation, as well as energy metabolism, were significantly higher in the RHS soil sample (*t*-test, corrected *p* < 0.01), while bacteria from the diseased soil samples had more functions related to membrane transport and second metabolite biosynthesis. At level 3, peroxisome, protein processing in the endoplasmic reticulum, and photosynthesis were significantly higher in RHS (*p* < 0.01), while the phosphotransferase system, tropane, piperidine, and pyridine alkaloid biosynthesis were in higher proportions in RPS (Figure 7A). The pathways related to the phosphotransferase system were significantly enhanced in the NRPS sample, while pantothenate and CoA biosynthesis, peptidoglycan biosynthesis, and fatty acid biosynthesis functions were obviously increased in the NRHS (Figure 7B). Functions related to antioxidant capacity were higher in the soil samples of *Verticillium* wilt-affected plants without a significant difference. The biofilm-forming function was significantly increased in bacteria from the rhizosphere soil compartments (Figure 7C). In addition, the trophic modes of the *C. coggygria* soil microbiome were dominated by saprotrophs, followed by pathotrophs and symbiotrophs (Supplementary Figure S9).

## Discussion

The occurrence of soil-borne diseases is closely related to the imbalance of the soil microecosystem; therefore, understanding the variability of the rhizosphere-associated soil microbiome is of great importance in plant health maintenance (Zheng et al., 2022). Rhizosphere-associated soil microbiome has been previously observed on a series of crops and plants (Dudenhöffer et al., 2016; Zhou et al., 2021). In order to determine whether the soil microbiome functioned to sustain *Cotinus coggygria* health, we investigated the microbial diversity, community composition, biomarker species, and interaction network of the rhizosphere and non-rhizosphere soil samples of *Verticillium* wilt-affected and healthy *C. coggygria* in an urban area of Beijing, China. Results indicated that the bacterial Shannoneven, Shannon, and InvSimpson indices in the rhizosphere soil of *Verticillium* wilt-affected *C. coggygria* exhibited a significant increase in comparison to those observed in healthy plants. These findings were consistent with the previous study, as the alpha diversity indices, including Chao1 and Shannon index, were significantly higher in root-rotted samples of areca nut palm (Li et al., 2021). However, the Shannon and Simpson indices of soil samples obtained from healthy cotton plants exhibited higher values compared to those derived from diseased plants (Wei et al., 2021). The fungal diversity indices, including Sobs, Chao, Ace, Shannoneven, and Shannon indices, exhibited a distinct reduction in the rhizosphere soil of diseased samples, which may be related to a decrease in species richness and an increase in abundance of some specific fungi (Li et al., 2023).

Plants select and shape the rhizosphere-associated soil microbiome, stimulating or repressing certain members of the indigenous microbial communities, which can act as the first defense line against soil-borne pathogens through diverse mechanisms (Berg et al., 2017; Fernández-González et al., 2020). In a comparison of the taxon abundance of soil microbiome across different soil samples, a significant reduction in specific bacteria such as Acidobacteria and Gemmatimonadetes was observed in the rhizosphere of *Verticillium* wilt-affected plants. This finding aligns with the recent research that the decline of Acidobacteria is associated with wilt development in olive (Fernández-González et al., 2020) and cotton (Wei et al., 2021), also caused by *V. dahliae*. The relative abundance of Gemmatimonadetes in the rhizosphere exhibited a negative correlation with *V. dahliae* infection, as documented by Fernández-González et al. (2020).

In this study, the genera *Arthrobacter*, *Bacillus,* and *Trichoderma* exhibited evident abundance in healthy soil samples; the phenomenon may involve the enrichment of beneficial microbes (Andric et al., 2020). *Trichoderma* was previously reported to promote plant growth or elicit disease resistance through the production of bioactive substances, regulation of related defensive pathways, or competition for space and nutrients with pathogens (Wei et al., 2019; Zhou et al., 2021). However, the relative abundance of *Streptomyces* was found to be increased in the *Verticillium* wilt-affected *C. coggygria*, which may be related to the recruitment of antagonistic microbes driven by diseased host plants (Yin et al., 2021), since *Streptomyces* usually produce secondary metabolites with biological activities and have been isolated from soil to inhibit phytopathogen growth (Barka et al., 2016; Takahashi and Nakashima, 2018). These were in accordance with the results of rhizosphere soil studies in rusty root-affected Ginseng (Wei et al., 2020) and *Fusarium* wilt-infected watermelon (Meng et al., 2019). The increased abundance of *Fusarium* in diseased plants may be associated with the occurrence and aggravation of *Verticillium* wilt in *C. coggygria*, agreeing with previous research that *Fusarium* contributed to the exacerbation of grapevine trunk disease (Li et al., 2023) and *Zanthoxylum bungeanum* root rot (Liao et al., 2022).

In-depth analyses of co-occurrence networks within microbial communities may facilitate the elucidation of the functional roles of microbiota and ascertain the stability of the ecosystem (Barberán et al., 2011; Fernández-González et al., 2020). In this study, certain genera, such as *Bacillus*, *Streptomyces*, *Solirubrobacter,* and *Sphingomonas,* exhibited higher clustering coefficients and occupied pivotal positions within the bacterial ecology. These keystone taxa frequently co-occur with other microbes and potentially play primary roles in microbial ecosystems (Agler et al., 2016; Banerjee et al., 2016, 2018). The enhanced tolerance to pathogens may be related to the intricate interaction network in soil ecology, implying that pathogen suppression is driven by a microbial consortium rather than individual microorganisms (Gómez et al., 2017; Zhou et al., 2021). The bacterial network illustrated higher network density and more edge numbers in diseased soil samples, while those were particularly pronounced in the fungal communities. Certain pathogenic or harmful fungi may interact with each other to exacerbate the disease severity of *Verticillium* wilt. For example, the genus *Fusarium* acted as the keystone taxon in the diseased network, exhibiting the highest clustering coefficient values and displaying positive correlations with 29 genera. The microbial community may respond to the perturbation and increase resistance against pathogen infection through more efficient information dissemination (Banerjee et al., 2018).

The rhizosphere-associated soil microbiome primarily referred to the microorganisms residing in soil particles that were closely related to the *C. coggygria* roots. Soil ecological niche-mediated microbiome variability has been demonstrated in different plant species. Similar to previous investigations conducted on other plants such as potato (Shi et al., 2019), rice (Edwards et al., 2015), and ficus trees (Liu et al., 2022), the rhizosphere soil was more likely to be influenced by root exudates, exhibiting distinct niche variability, which showed significant enrichment of *Bacillus* and *Mycobacterium*. Together, our findings demonstrated that *C. coggygria* possessed the ability to modulate the rhizosphere-associated soil microbiome under different plant health statuses. The healthy plants selectively recruited a consortium of beneficial microbes such as *Arthrobacter*, *Bacillus*, *and Trichoderma*; these microbial species may exert their effects on the establishment of a favorable rhizosphere micro-ecological environment through positive interaction (Berendsen et al., 2018). Understanding the correlation between plant health and soil microbiome holds the potential for manipulating soil microbiome to enhance plant health and manage plant disease (Kwak et al., 2018; Pascale et al., 2020). Further studies will focus on the isolation and screening of the enriched beneficial species or keystone taxa, and on the evaluation of their consistency in plant growth promotion and disease suppression abilities.

## Conclusion

The composition and structure patterns of the rhizosphere-associated soil microbiome in *C. coggygria* were not well understood in the forest ecosystem, nor were those from different plant health status or soil compartments. Our study provides a comprehensive study of the rhizosphere-associated soil microbiome variability of *Verticillium* wilt-affected *C. coggygria* in Beijing, China. The microbial diversity, community composition, and abundance of certain microbial taxa varied in accordance with the plants’ healthy status and soil compartments. The disease status of *Verticillium* wilt strengthened the microbial nodes and their interactions in the co-occurrence network, especially the fungal community. The results improved our understanding of the association between the rhizosphere-associated soil microbiome and *Verticillium* wilt occurrence in the *C. coggygria* plant, which has important implications for soil-borne disease management in forest tree species in future.

## Data availability statement

The datasets presented in this study can be found in online repositories. The names of the repository/repositories and accession number(s) can be found in the article/Supplementary material.

## Author contributions

JZ: Data curation, Investigation, Project administration, Writing – original draft, Writing – review & editing. YC: Investigation, Methodology, Validation, Writing – review & editing. NJ: Investigation, Methodology, Validation, Writing – review & editing. GQ: Investigation, Methodology, Validation, Writing – review & editing. WQ: Project administration, Supervision, Validation, Writing – review & editing.

## Footnotes

^1^ http://www.arb-silva.de

^2^ http://unite.ut.ee/index.php

^3^ https://cloud.majorbio.com/

^4^ http://huttenhower.sph.harvard.edu/lefse/
