# Supplementary material for: Rhizosphere-associated soil microbiome variability in Verticillium wilt-affected Cotinus coggygria
Source: Front Microbiol. 2024 Jan 5;14:1279096. doi: 10.3389/fmicb.2023.1279096 (PMC10797040; doi:10.3389/fmicb.2023.1279096)
Supplement: Supplementary file 1 [file Data_Sheet_1.pdf]

Supplementary Table S1 Relative abundance of the dominant microbial taxa in *Cotinus coggygria* soil microbiome under different conditions

Taxon		RHS	RPS	NRHS	NRPS	LS
Bacteria	Actinobacteria	0.33±0.02 b	0.35±0.02 b	0.42±0.05 a	0.42±0.02 a	0.38±0.01 ab
phylum	Proteobacteria	0.23±0.01 ab	0.24±0.01 a	0.22±0.04 ab	0.19±0.02 b	0.20±0.02 ab
	Acidobacteria	0.14±0.02 a	0.13±0.02 a	0.13±0.07 a	0.13±0.03 a	0.17±0.03 a
	Chloroflexi	0.07±0.01 b	0.10±0.00 a	0.08±0.02 b	0.11±0.01 a	0.10±0.01 a
	Firmicutes	0.08±0.01 a	0.02±0.00 b	0.02±0.00 b	0.03±0.01 b	0.02±0.01 b
	Gemmatimonadota	0.04±0.00 a	0.03±0.00 a	0.03±0.00 a	0.04±0.00 a	0.03±0.00 a
Bacteria	Actinobacteria	0.21±0.01a	0.20±0.01 a	0.22±0.02 a	0.15±0.00 b	0.15±0.01 b
class	Alphaproteobacteria	0.16±0.00 ab	0.17±0.01 a	0.16±0.03 ab	0.14±0.01 b	0.15±0.01 ab
	Thermoleophilia	0.08±0.02 c	0.11±0.01 c	0.15±0.03 b	0.20±0.02 a	0.15±0.02 b
	Vicinamibacteria	0.09±0.01 a	0.08±0.01 a	0.08±0.05 a	0.08±0.02 a	0.10±0.01 a
	Gammaproteobacteria	0.07±0.01 a	0.07±0.00 ab	0.06±0.01 ab	0.04±0.00 b	0.06±0.01 b
Fungi	Ascomycota	0.79±0.01 a	0.64±0.15 a	0.73±0.02 a	0.67±0.15 a	0.77±0.03 a
phylum	Basidiomycota	0.14±0.02 b	0.35±0.15 a	0.16±0.01 b	0.20±0.11 ab	0.12±0.02 bc
	Mortierellomycota	0.04±0.01 b	0.01±0.00 c	0.06±0.01 ab	0.08±0.03 a	0.07±0.02 a
Fungi	Dothideomycetes	0.41±0.02 a	0.53±0.17 a	0.14±0.01 b	0.15±0.07 b	0.15±0.05 b

class	Sordariomycetes	0.19 ±0.05 a	0.04 ±0.01 b	0.25 ±0.01 a	0.30 ±0.10 a	0.27 ±0.09 a
	Eurotiomycetes	0.10 ±0.03 b	0.04 ±0.01 c	0.17 ±0.02 a	0.15 ±0.02 ab	0.16 ±0.06 ab
	Agaricomycetes	0.09 ±0.03 b	0.34 ±0.15 a	0.02 ±0.00 c	0.03 ±0.01 c	0.02 ±0.01 c
	Tremellomycetes	0.05 ±0.01 b	0.01 ±0.00 c	0.14 ±0.01 a	0.17 ±0.11 a	0.10 ±0.02 ab

Values were expressed as the mean values ± standard error. Different letters in the same row indicated significant differences among different soil samples according to the LSD test ($P < 0.05$). RHS: rhizosphere soil of healthy plants; RPS: rhizosphere soil of *Verticillium* wilt-affected plants; NRHS: non-rhizosphere soil of healthy plants; NRPS: non-rhizosphere soil of *Verticillium* wilt-affected plants; LS: bulk soil in the *C. coggygria* forest.

Supplementary Table S2 Topological indices of soil bacterial and fungal co-occurrence networks in *Verticillium* wilt-affected and healthy *Cotinus coggygria*

Sample numbers	Topological indices						
	Degrees	Degree centrality	Closeness centrality	Network density	Total edges	Positive edges (Percent)	Negative edges (Percent)
BHS	13.12 ±8.56 a	0.13 ±0.09 a	0.33 ±0.06 a	0.133	656	450 (68.6%)	206 (31.4%)
BPS	20.85 ±12.41 a	0.21 ±0.13 a	0.38 ±0.13 a	0.213	1032	546 (52.9%)	486 (47.1%)
FHS	14.40 ±9.05 a	0.14 ±0.09 a	0.33 ±0.07 a	0.145	720	450 (62.5%)	270 (37.5%)
FPS	23.92 ±15.87 a	0.25 ±0.17 a	0.44 ±0.10 a	0.249	1160	981 (84.6%)	179 (15.4%)

To gain a deeper insight into the network interaction, the soil samples collected from rhizosphere and non-rhizosphere compartments of *Cotinus coggygria* under same healthy status were merged. Different letters in the same column indicated significant differences between BHS and BPS networks or FHS and FPS networks according to the LSD test ($P < 0.05$). BHS: bacterial network of heathy plants; BPS: bacterial network of *Verticillium* wilt-affected plants; FHS: fungal network of healthy plants; FPS: fungal network of *Verticillium* wilt-affected plants.

Supplementary Table S3 The topological indices of co-occurrence network for bacteria in the soil samples of healthy *Cotinus coggygria*

Label	Degree centrality	Closeness centrality	Betweenness centrality	Degree	Clustering	Phylum
norank_c__JG30-KF-CM66	0.061	0.322	0.053	6	0.333	Chloroflexi
Parafrigoribacterium	0.152	0.382	0.095	15	0.324	Actinobacteriota
norank_f__Methyloligellaceae	0.030	0.256	0.059	3	0.333	Proteobacteria
norank_f__Xanthobacteraceae	0.081	0.322	0.056	8	0.393	Proteobacteria
Nordella	0.051	0.301	0.009	5	0.600	Proteobacteria
Microlunatus	0.051	0.301	0.009	5	0.600	Actinobacteriota
Terrimonas	0.061	0.307	0.020	6	0.600	Bacteroidota
Skermanella	0.131	0.367	0.016	13	0.679	Proteobacteria
Sphingomonas	0.131	0.367	0.016	13	0.679	Proteobacteria
Virgisporangium	0.192	0.390	0.022	19	0.626	Actinobacteriota
Iamia	0.242	0.390	0.031	24	0.594	Actinobacteriota
Conexibacter	0.263	0.425	0.032	26	0.609	Actinobacteriota
Streptomyces	0.101	0.332	0.006	10	0.644	Actinobacteriota
Allorhizobium-Neorhizobium-Pararhizobium-Rhizobium	0.101	0.332	0.006	10	0.644	Proteobacteria
Bacillus	0.081	0.346	0.009	8	0.500	Firmicutes
norank_f__Gemmatimonadaceae	0.131	0.361	0.022	13	0.513	Gemmatimonadota
Bradyrhizobium	0.172	0.391	0.036	17	0.441	Proteobacteria
Gemmatimonas	0.192	0.394	0.030	19	0.532	Gemmatimonadota
Mesorhizobium	0.192	0.394	0.030	19	0.532	Proteobacteria
Reyranella	0.212	0.423	0.128	21	0.381	Proteobacteria
Rubrobacter	0.101	0.364	0.016	10	0.756	Actinobacteriota
Dongia	0.111	0.367	0.015	11	0.600	Proteobacteria

norank_f__TRA3-20	0.101	0.364	0.016	10	0.756	Proteobacteria
MND1	0.101	0.364	0.016	10	0.756	Proteobacteria
Haliangium	0.152	0.376	0.010	15	0.581	Myxococcota
Mycobacterium	0.152	0.376	0.010	15	0.581	Actinobacteriota
norank_c__Alphaproteobacteria	0.111	0.345	0.058	11	0.400	Proteobacteria
norank_o__S085	0.030	0.275	0.000	3	1.000	Chloroflexi
norank_o__Azospirillales	0.212	0.344	0.025	21	0.538	Proteobacteria
norank_o__11-24	0.121	0.301	0.004	12	0.621	Acidobacteriota
norank_f__Vicinamibacteraceae	0.121	0.333	0.012	12	0.667	Acidobacteriota
norank_o__Elsterales	0.020	0.270	0.000	2	1.000	Proteobacteria
norank_o__Rokubacteriales	0.091	0.298	0.000	9	1.000	Methylomirabilota
Candidatus_Alysiosphaera	0.222	0.376	0.022	22	0.584	Proteobacteria
norank_o__Microtrichales	0.222	0.376	0.022	22	0.584	Actinobacteriota
norank_o__IMCC26256	0.273	0.398	0.015	27	0.670	Actinobacteriota
Solirubrobacter	0.273	0.398	0.015	27	0.670	Actinobacteriota
Blastococcus	0.273	0.398	0.015	27	0.670	Actinobacteriota
norank_f__67-14	0.273	0.398	0.015	27	0.670	Actinobacteriota
norank_f__Ilumatobacteraceae	0.273	0.398	0.015	27	0.670	Actinobacteriota
Arthrobacter	0.091	0.298	0.000	9	1.000	Actinobacteriota
norank_f__A4b	0.273	0.398	0.015	27	0.670	Chloroflexi
Pseudolabrys	0.152	0.324	0.002	15	0.895	Proteobacteria
norank_f__Geminicoccaceae	0.152	0.324	0.002	15	0.895	Proteobacteria
unclassified_o__Gaiellales	0.162	0.375	0.004	16	0.875	Actinobacteriota
norank_c__Thermoleophilia	0.253	0.355	0.013	25	0.640	Actinobacteriota
norank_o__Gaiellales	0.253	0.355	0.013	25	0.640	Actinobacteriota
norank_o__Rhizobiales	0.202	0.368	0.019	20	0.574	Proteobacteria

Microvirga	0.222	0.368	0.011	22	0.710	Proteobacteria
Rhodanobacter	0.222	0.368	0.011	22	0.710	Proteobacteria
norank_f__Gemmataceae	0.111	0.349	0.013	11	0.655	Planctomycetota
norank_f__Blastocatellaceae	0.202	0.368	0.019	20	0.574	Acidobacteriota
Candidatus_Udaeobacter	0.202	0.368	0.019	20	0.574	Verrucomicrobiota
norank_o__Vicinamibacterales	0.101	0.343	0.010	10	0.711	Acidobacteriota
Gaiella	0.253	0.390	0.024	25	0.627	Actinobacteriota
Microbacterium	0.313	0.407	0.018	31	0.585	Actinobacteriota
Lysobacter	0.313	0.407	0.018	31	0.585	Proteobacteria
Marmoricola	0.313	0.407	0.018	31	0.585	Actinobacteriota
Nocardioides	0.313	0.407	0.018	31	0.585	Actinobacteriota
CL500-29_marine_group	0.313	0.407	0.018	31	0.585	Actinobacteriota
Nitrospira	0.061	0.294	0.000	6	1.000	Nitrospirota
Actinoplanes	0.172	0.376	0.007	17	0.750	Actinobacteriota
Pedomicrobium	0.172	0.376	0.007	17	0.750	Proteobacteria
Krasilnikovia	0.192	0.398	0.073	19	0.614	Actinobacteriota
norank_c__bacteriap25	0.263	0.384	0.037	26	0.551	Myxococcota
RB41	0.091	0.351	0.064	9	0.472	Acidobacteriota
unclassified_f__Micromonosporaceae	0.051	0.333	0.005	5	0.700	Actinobacteriota
norank_f__Microscillaceae	0.061	0.349	0.080	6	0.267	Bacteroidota
norank_o__PLTA13	0.051	0.298	0.000	5	0.900	Proteobacteria
norank_c__TK10	0.051	0.298	0.000	5	0.900	Chloroflexi
norank_f__Entotheonellaceae	0.061	0.304	0.001	6	0.733	Entotheonellaeota
Steroidobacter	0.061	0.310	0.025	6	0.333	Proteobacteria
Bryobacter	0.061	0.301	0.018	6	0.467	Acidobacteriota
norank_f__Chitinophagaceae	0.091	0.314	0.051	9	0.333	Bacteroidota

norank_o__Subgroup_7	0.091	0.346	0.002	9	0.778	Acidobacteriota
Ellin6067	0.081	0.352	0.045	8	0.750	Proteobacteria
Halocella	0.061	0.319	0.045	6	0.533	Halanaerobiaeota
norank_c__MB-A2-108	0.051	0.354	0.073	5	0.500	Actinobacteriota
Rhodoplanes	0.081	0.274	0.031	8	0.500	Proteobacteria
Subgroup_10	0.061	0.239	0.004	6	0.467	Acidobacteriota
Phenylobacterium	0.051	0.219	0.000	5	0.800	Proteobacteria
Ellin6055	0.040	0.219	0.000	4	1.000	Proteobacteria
norank_o__Subgroup_17	0.081	0.274	0.031	8	0.500	Acidobacteriota
Hydrogenispora	0.030	0.217	0.000	3	1.000	Firmicutes
norank_c__Gitt-GS-136	0.121	0.333	0.045	12	0.409	Chloroflexi
norank_o__C0119	0.121	0.333	0.045	12	0.409	Chloroflexi
Kribbella	0.071	0.300	0.040	7	0.381	Actinobacteriota
norank_f__SC-I-84	0.030	0.236	0.001	3	0.667	Proteobacteria
norank_f__Roseiflexaceae	0.040	0.258	0.004	4	0.500	Chloroflexi
Acidibacter	0.091	0.319	0.022	9	0.444	Proteobacteria
norank_f__JG30-KF-CM45	0.010	0.237	0.000	1	0.000	Chloroflexi
unclassified_f__Comamonadaceae	0.040	0.244	0.020	4	0.333	Proteobacteria
Nakamurella	0.010	0.196	0.000	1	0.000	Actinobacteriota
norank_c__KD4-96	0.091	0.310	0.012	9	0.611	Chloroflexi
norank_c__S0134_terrestrial_group	0.040	0.320	0.011	4	0.500	Gemmatimonadota
Paenibacillus	0.030	0.206	0.040	3	0.333	Firmicutes
unclassified_k__norank_d__Bacteria	0.020	0.172	0.000	2	1.000	unclassified_k__norank_d__Bacteria
Thermostaphylospora	0.020	0.172	0.000	2	1.000	Actinobacteriota
Pseudonocardia	0.101	0.336	0.057	10	0.467	Actinobacteriota

Pseudaminobacter	0.081	0.305	0.010	8	0.571	Proteobacteria

To gain a deeper insight into the interaction among bacteria from soil samples of healthy plants, the bacteria in the rhizosphere (RHS) and non-rhizosphere (NRHS) soils were merged (BHS) to construct the co-occurrence network. A connection denoted a strong (Spearman's ρ>0.8 or <-0.8) and significant (P< 0.05) correlation.

Supplementary Table S4 The topological indices of co-occurrence network for bacteria in the soil samples of *Verticillium* wilt-affected *Cotinus coggygria*

Lable	Degree Centrality	Closeness Centrality	Betweenness Centrality	Degree	Clustering	Phylum
Rhodoplanes	0.010	0.269	0.000	1	0.000	Proteobacteria
norank_f__Roseiflexaceae	0.102	0.379	0.021	10	0.622	Chloroflexi
Microbispora	0.133	0.392	0.047	13	0.577	Actinobacteriota
Dongia	0.214	0.440	0.020	21	0.505	Proteobacteria
Virgisporangium	0.214	0.440	0.020	21	0.505	Actinobacteriota
Rhodomicrobium	0.235	0.459	0.042	23	0.443	Proteobacteria
Microlunatus	0.173	0.437	0.013	17	0.485	Actinobacteriota
Bryobacter	0.071	0.324	0.013	7	0.619	Acidobacteriota
unclassified_f__Gemmatimonadaceae	0.173	0.437	0.013	17	0.485	Gemmatimonadota
norank_f__norank_o__norank_c__Gitt-GS-136	0.214	0.440	0.020	21	0.505	Chloroflexi
Paenibacillus	0.184	0.449	0.020	18	0.471	Firmicutes
Steroidobacter	0.204	0.444	0.018	20	0.532	Proteobacteria
Mycobacterium	0.245	0.489	0.050	24	0.471	Actinobacteriota
Pseudolabrys	0.082	0.293	0.023	8	0.571	Proteobacteria
norank_f__norank_o__11-24	0.122	0.369	0.075	12	0.364	Acidobacteriota
norank_f__norank_o__norank_c__KD4-96	0.082	0.293	0.023	8	0.571	Chloroflexi
norank_f__norank_o__S085	0.245	0.489	0.050	24	0.471	Chloroflexi
norank_f__norank_o__norank_c__TK10	0.092	0.356	0.021	9	0.528	Chloroflexi
norank_f__norank_o__PLTA13	0.071	0.356	0.000	7	0.905	Proteobacteria
Krasilnikovia	0.173	0.420	0.008	17	0.603	Actinobacteriota
Allorhizobium-Neorhizobium-Pararhizobium-Rhizobium	0.235	0.430	0.011	23	0.545	Proteobacteria

Ellin6067	0.337	0.462	0.030	33	0.445	Proteobacteria
norank_f__norank_o__Saccharimonadales	0.265	0.444	0.017	26	0.548	Patescibacteria
norank_f__JG30-KF-CM45	0.224	0.433	0.021	22	0.455	Chloroflexi
norank_f__AKYG1722	0.224	0.433	0.021	22	0.455	Chloroflexi
Blastococcus	0.031	0.301	0.000	3	1.000	Actinobacteriota
Conexibacter	0.010	0.257	0.000	1	0.000	Actinobacteriota
Actinoplanes	0.255	0.457	0.012	25	0.540	Actinobacteriota
Streptomyces	0.306	0.483	0.030	30	0.464	Actinobacteriota
Haliangium	0.184	0.437	0.007	18	0.529	Myxococcota
Bradyrhizobium	0.296	0.481	0.021	29	0.498	Proteobacteria
Kribbella	0.184	0.417	0.003	18	0.667	Actinobacteriota
norank_f__Xanthobacteraceae	0.184	0.417	0.003	18	0.667	Proteobacteria
norank_f__Gemmataceae	0.235	0.413	0.007	23	0.609	Planctomycetota
SWB02	0.245	0.462	0.008	24	0.710	Proteobacteria
norank_f__norank_o__Chloroplast	0.245	0.462	0.008	24	0.710	Cyanobacteria
norank_f__Methyloligellaceae	0.245	0.462	0.008	24	0.710	Proteobacteria
Sporosarcina	0.316	0.483	0.023	31	0.503	Firmicutes
norank_f__A4b	0.306	0.454	0.012	30	0.632	Chloroflexi
Candidatus_Udaeobacter	0.306	0.454	0.012	30	0.632	Verrucomicrobiota
norank_f__norank_o__IMCC26256	0.316	0.489	0.018	31	0.639	Actinobacteriota
Iamia	0.306	0.454	0.012	30	0.632	Actinobacteriota
Dactylosporangium	0.255	0.472	0.008	25	0.630	Actinobacteriota
Gemmatimonas	0.255	0.472	0.008	25	0.630	Gemmatimonadota
Mesorhizobium	0.255	0.472	0.008	25	0.630	Proteobacteria
Candidatus_Alysiosphaera	0.143	0.422	0.014	14	0.692	Proteobacteria
Rubrobacter	0.255	0.472	0.008	25	0.630	Actinobacteriota

Pseudonocardia	0.235	0.417	0.003	23	0.652	Actinobacteriota
Phenylobacterium	0.398	0.472	0.015	39	0.601	Proteobacteria
norank_f__Blastocatellaceae	0.459	0.486	0.019	45	0.580	Acidobacteriota
Vicinamibacter	0.459	0.486	0.019	45	0.580	Acidobacteriota
Acidibacter	0.357	0.447	0.005	35	0.708	Proteobacteria
Actinophytocola	0.357	0.447	0.005	35	0.708	Actinobacteriota
norank_f__norank_o__norank_c__Alphaproteobacteria	0.357	0.447	0.005	35	0.708	Proteobacteria
Ellin6055	0.398	0.481	0.017	39	0.613	Proteobacteria
Actinomadura	0.306	0.475	0.020	30	0.552	Actinobacteriota
norank_f__norank_o__norank_c__MB-A2-108	0.306	0.475	0.020	30	0.552	Actinobacteriota
norank_f__norank_o__C0119	0.347	0.437	0.003	34	0.781	Chloroflexi
Parafrigoribacterium	0.347	0.437	0.003	34	0.781	Actinobacteriota
Reyranella	0.347	0.437	0.003	34	0.781	Proteobacteria
Sphingomonas	0.398	0.464	0.007	39	0.692	Proteobacteria
norank_f__norank_o__norank_c__norank_RCP2-54	0.347	0.437	0.003	34	0.781	RCP2-54
norank_f__Gemmatimonadaceae	0.347	0.437	0.003	34	0.781	Gemmatimonadota
norank_f__67-14	0.347	0.437	0.003	34	0.781	Actinobacteriota
norank_f__norank_o__norank_c__Thermoleophilia	0.347	0.437	0.003	34	0.781	Actinobacteriota
norank_f__Sandaracinaceae	0.347	0.486	0.030	34	0.640	Myxococcota
norank_f__norank_o__Rokubacteriales	0.071	0.343	0.020	7	0.619	Methylomirabilota
norank_f__norank_o__norank_c__JG30-KF-CM66	0.082	0.383	0.005	8	0.750	Chloroflexi
Nocardioides	0.102	0.363	0.000	10	0.822	Actinobacteriota
Bacillus	0.286	0.452	0.007	28	0.638	Firmicutes
norank_f__norank_o__Azospirillales	0.327	0.449	0.005	32	0.742	Proteobacteria
norank_f__Ilumatobacteraceae	0.327	0.449	0.005	32	0.742	Actinobacteriota
Solirubrobacter	0.327	0.449	0.005	32	0.742	Actinobacteriota

norank_f__Micromonosporaceae	0.286	0.452	0.007	28	0.638	Actinobacteriota
Nordella	0.357	0.457	0.004	35	0.760	Proteobacteria
norank_f__norank_o__Gaiellales	0.357	0.457	0.004	35	0.760	Actinobacteriota
Gaiella	0.357	0.457	0.004	35	0.760	Actinobacteriota
unclassified_o__Gaiellales	0.357	0.457	0.004	35	0.760	Actinobacteriota
norank_f__Geminicoccaceae	0.357	0.457	0.004	35	0.760	Proteobacteria
Nitrospira	0.061	0.335	0.001	6	0.800	Nitrospirota
Microvirga	0.276	0.405	0.010	27	0.860	Proteobacteria
norank_f__norank_o__norank_c__bacteriap25	0.276	0.405	0.010	27	0.860	Myxococcota
norank_f__norank_o__Rhizobiales	0.082	0.349	0.000	8	1.000	Proteobacteria
norank_f__norank_o__Elsterales	0.010	0.015	0.000	1	0.000	Proteobacteria
norank_f__SC-I-84	0.020	0.023	0.000	2	0.000	Proteobacteria
norank_f__TRA3-20	0.020	0.023	0.000	2	0.000	Proteobacteria
MND1	0.010	0.015	0.000	1	0.000	Proteobacteria
unclassified_f__Micromonosporaceae	0.010	0.010	0.000	1	0.000	Actinobacteriota
norank_f__Microscillaceae	0.010	0.010	0.000	1	0.000	Bacteroidota
Pedomicrobium	0.051	0.227	0.000	5	1.000	Proteobacteria
norank_f__Vicinamibacteraceae	0.051	0.227	0.000	5	1.000	Acidobacteriota
norank_f__norank_o__Vicinamibacterales	0.061	0.273	0.005	6	0.800	Acidobacteriota
norank_f__norank_o__Subgroup_7	0.082	0.230	0.004	8	0.464	Acidobacteriota
Arthrobacter	0.010	0.010	0.000	1	0.000	Actinobacteriota
norank_f__norank_o__Microtrichales	0.010	0.010	0.000	1	0.000	Actinobacteriota
RB41	0.051	0.271	0.010	5	0.600	Acidobacteriota
Marmoricola	0.051	0.271	0.010	5	0.600	Actinobacteriota
norank_f__norank_o__Subgroup_17	0.031	0.214	0.000	3	1.000	Acidobacteriota
norank_f__Entotheonellaceae	0.031	0.284	0.000	3	1.000	Entotheonellaeota

To gain a deeper insight into the interactions among bacteria from soil samples of *Verticillium* wilt-affected plants, the bacteria in the rhizosphere (RPS) and non-rhizosphere (NRPS) soils were merged (BPS) to construct the co-occurrence network. A connection denoted a strong (Spearman's ρ>0.8 or <-0.8) and significant (P< 0.05) correlation.

Supplementary Table S5 The topological indices of co-occurrence network for fungi in the soil samples of healthy *C. coggygria*

Genus	Degree Centrality	Closeness Centrality	Betweenness Centrality	Degree	Clustering	Phylum
unclassified_o__Orbiliales	0.040	0.166	0.007	4	0.667	Ascomycota
unclassified_c__Orbiliomycetes	0.020	0.143	0.000	2	1.000	Ascomycota
Lepiota	0.061	0.167	0.014	6	0.333	Basidiomycota
Bradymyces	0.040	0.197	0.021	4	0.667	Ascomycota
unclassified_c__Eurotiomycetes	0.040	0.197	0.021	4	0.667	Ascomycota
unclassified_k__Fungi	0.030	0.196	0.008	3	0.667	unclassified_k__Fungi
Neocosmospora	0.030	0.196	0.008	3	0.667	Ascomycota
unclassified_o__Chaetothyriales	0.051	0.240	0.133	5	0.200	Ascomycota
Fusarium	0.081	0.302	0.176	8	0.464	Ascomycota
Aplosporella	0.030	0.270	0.027	3	0.333	Ascomycota
Oidiodendron	0.040	0.281	0.018	4	0.333	Ascomycota
Desmazierella	0.061	0.286	0.032	6	0.333	Ascomycota
Schizothecium	0.061	0.277	0.039	6	0.467	Ascomycota
Paraphoma	0.061	0.312	0.014	6	0.467	Ascomycota
unclassified_f__Chaetomiaceae	0.061	0.312	0.014	6	0.467	Ascomycota
Exophiala	0.030	0.268	0.000	3	1.000	Ascomycota
Leohumicola	0.040	0.277	0.000	4	0.833	Ascomycota
Paraconiothyrium	0.101	0.347	0.048	10	0.378	Ascomycota
Phialosimplex	0.051	0.325	0.018	5	0.500	Ascomycota
unclassified_o__Pezizales	0.091	0.313	0.021	9	0.500	Ascomycota
unclassified_o__Capnodiales	0.101	0.268	0.026	10	0.556	Ascomycota
unclassified_o__Auriculariales	0.111	0.375	0.249	11	0.418	Basidiomycota
Cephalotrichum	0.071	0.301	0.007	7	0.714	Ascomycota

unclassified_f__Agaricaceae	0.091	0.303	0.026	9	0.556	Basidiomycota
Olpidium	0.111	0.300	0.037	11	0.527	Olpidiomycota
Mycoarthris	0.232	0.436	0.068	23	0.569	Ascomycota
Holtermanniella	0.232	0.436	0.068	23	0.569	Basidiomycota
Cladophialophora	0.232	0.436	0.068	23	0.569	Ascomycota
Didymella	0.081	0.249	0.002	8	0.714	Ascomycota
unclassified_f__Pannariaceae	0.071	0.243	0.001	7	0.714	Ascomycota
unclassified_o__Hypocreales	0.061	0.246	0.000	6	0.933	Ascomycota
Penicillium	0.061	0.246	0.000	6	0.933	Ascomycota
unclassified_f__Nectriaceae	0.040	0.237	0.000	4	0.833	Ascomycota
Sagenomella	0.030	0.240	0.000	3	1.000	Ascomycota
Pseudogymnoascus	0.040	0.253	0.001	4	0.667	Ascomycota
Cyphellophora	0.061	0.287	0.020	6	0.467	Ascomycota
Minimedusa	0.040	0.289	0.015	4	0.333	Basidiomycota
Chaetopyrena	0.081	0.312	0.019	8	0.500	Ascomycota
Talaromyces	0.030	0.273	0.000	3	1.000	Ascomycota
Infundichalara	0.263	0.372	0.027	26	0.511	Ascomycota
Fusicolla	0.263	0.372	0.027	26	0.511	Ascomycota
Dictyosporium	0.030	0.273	0.000	3	1.000	Ascomycota
Pseudombrophila	0.091	0.306	0.005	9	0.750	Ascomycota
unclassified_Chytridiomycota	0.242	0.378	0.025	24	0.442	Chytridiomycota
unclassified_f__Hyaloscyphaceae	0.192	0.361	0.037	19	0.491	Ascomycota
Phaeosphaeria	0.111	0.346	0.004	11	0.582	Ascomycota
Harmoniella	0.222	0.346	0.010	22	0.567	Ascomycota
Wardomyces	0.222	0.346	0.010	22	0.567	Ascomycota
unclassified_f__Didymosphaeriaceae	0.222	0.346	0.010	22	0.567	Ascomycota

unclassified_Basidiomycota	0.162	0.361	0.008	16	0.567	Basidiomycota
Gibberella	0.202	0.365	0.017	20	0.605	Ascomycota
Beauveria	0.010	0.226	0.000	1	0.000	Ascomycota
unclassified_f__Herpotrichiellaceae	0.051	0.291	0.020	5	0.400	Ascomycota
Acrocalymma	0.051	0.320	0.000	5	1.000	Ascomycota
unclassified_o__Agaricales	0.162	0.401	0.054	16	0.650	Basidiomycota
Clonostachys	0.313	0.416	0.033	31	0.497	Ascomycota
Metarhizium	0.162	0.374	0.014	16	0.667	Ascomycota
Trichoderma	0.162	0.374	0.014	16	0.667	Ascomycota
unclassified_o__Helotiales	0.313	0.416	0.033	31	0.497	Ascomycota
Robillarda	0.020	0.273	0.000	2	1.000	Ascomycota
Coniosporium	0.061	0.284	0.022	6	0.267	Ascomycota
Chaetomium	0.051	0.306	0.003	5	0.400	Ascomycota
Arthrographis	0.162	0.361	0.019	16	0.567	Ascomycota
unclassified_c__Agaricomycetes	0.162	0.361	0.019	16	0.567	Basidiomycota
Periconia	0.010	0.222	0.000	1	0.000	Ascomycota
unclassified_Rozellomycota	0.232	0.406	0.028	23	0.518	Rozellomycota
Sebacina	0.232	0.406	0.028	23	0.518	Basidiomycota
unclassified_Ascomycota	0.202	0.399	0.030	20	0.542	Ascomycota
Cystofilobasidium	0.202	0.399	0.030	20	0.542	Basidiomycota
Auxarthron	0.152	0.382	0.003	15	0.686	Ascomycota
Agaricus	0.222	0.390	0.006	22	0.658	Basidiomycota
Knufia	0.222	0.390	0.006	22	0.658	Ascomycota
Phoma	0.222	0.390	0.006	22	0.658	Ascomycota
Tausonia	0.283	0.418	0.020	28	0.526	Basidiomycota
Bartalinia	0.303	0.423	0.044	30	0.457	Ascomycota

unclassified_o__Glomerellales	0.293	0.387	0.018	29	0.507	Ascomycota
unclassified_c__Tremellomycetes	0.232	0.404	0.011	23	0.585	Basidiomycota
unclassified_Mortierellomycota	0.172	0.388	0.015	17	0.566	Mortierellomycota
unclassified_c__Sordariomycetes	0.283	0.418	0.020	28	0.526	Ascomycota
Phialophora	0.172	0.404	0.044	17	0.713	Ascomycota
Naganishia	0.253	0.411	0.022	25	0.580	Basidiomycota
Cladosporium	0.253	0.411	0.022	25	0.580	Ascomycota
unclassified_o__Pleosporales	0.202	0.398	0.042	20	0.526	Ascomycota
Phialocephala	0.293	0.394	0.026	29	0.475	Ascomycota
Neonectria	0.182	0.360	0.004	18	0.673	Ascomycota
Lophotrichus	0.313	0.393	0.019	31	0.488	Ascomycota
unclassified_o__Xylariales	0.242	0.404	0.008	24	0.638	Ascomycota
unclassified_o__Branch06	0.242	0.404	0.008	24	0.638	Ascomycota
Camarographium	0.242	0.404	0.008	24	0.638	Ascomycota
Mortierella	0.263	0.385	0.029	26	0.492	Mortierellomycota
Cylindrocarpon	0.232	0.374	0.016	23	0.545	Ascomycota
Aspergillus	0.172	0.355	0.004	17	0.654	Ascomycota
Preussia	0.232	0.374	0.016	23	0.545	Ascomycota
Dactylonectria	0.172	0.355	0.004	17	0.654	Ascomycota
Gonytrichum	0.101	0.330	0.003	10	0.733	Ascomycota
Bionectria	0.212	0.359	0.006	21	0.657	Ascomycota
unclassified_f__Didymellaceae	0.212	0.359	0.006	21	0.657	Ascomycota
Venturia	0.212	0.359	0.006	21	0.657	Ascomycota
Paraboeremia	0.192	0.368	0.018	19	0.608	Ascomycota
Solicoccozyma	0.192	0.368	0.018	19	0.608	Basidiomycota

To gain a deeper insight into the interaction among fungi from soil samples of healthy plants, the fungi in the rhizosphere (RHS) and

non-rhizosphere (NRHS) soils were merged (FHS) to construct the co-occurrence network. A connection denoted a strong (Spearman's ρ>0.8 or <-0.8) and significant (P< 0.05) correlation.

Supplementary Table S6 The topological indices of co-occurrence network for fungi in the soil samples of *Verticillium* wilt-affected *Cotinus coggygria*

Lable	Degree Centrality	Closeness Centrality	Betweenness Centrality	Degree	Clustering	Phylum
Russula	0.313	0.480	0.015	30	0.618	Basidiomycota
Neonectria	0.094	0.387	0.000	9	0.917	Ascomycota
Cladophialophora	0.094	0.403	0.035	9	0.667	Ascomycota
Naganishia	0.156	0.444	0.006	15	0.629	Basidiomycota
unclassified_f__Microascaceae	0.302	0.495	0.044	29	0.571	Ascomycota
Solicoccozyma	0.281	0.490	0.028	27	0.652	Basidiomycota
unclassified_Chytridiomycota	0.313	0.480	0.015	30	0.618	Chytridiomycota
Clonostachys	0.406	0.511	0.006	39	0.722	Ascomycota
Gibberella	0.406	0.511	0.006	39	0.722	Ascomycota
unclassified_f__Didymellaceae	0.406	0.511	0.006	39	0.722	Ascomycota
Cephaliophora	0.406	0.482	0.008	39	0.699	Ascomycota
Fusarium	0.469	0.527	0.014	45	0.644	Ascomycota
Metarhizium	0.469	0.527	0.014	45	0.644	Ascomycota
Knufia	0.333	0.482	0.008	32	0.762	Ascomycota
Schizothecium	0.333	0.482	0.008	32	0.762	Ascomycota
Pseudeurotium	0.333	0.482	0.008	32	0.762	Ascomycota
unclassified_Rozellomycota	0.448	0.516	0.006	43	0.720	Rozellomycota
unclassified_f__Thelephoraceae	0.448	0.516	0.006	43	0.720	Basidiomycota
unclassified_o__Hypocreales	0.448	0.516	0.006	43	0.720	Ascomycota
unclassified_k__Fungi	0.448	0.516	0.006	43	0.720	unclassified_k__Fungi
Humicola	0.448	0.516	0.006	43	0.720	Ascomycota

Phialemonium	0.448	0.516	0.006	43	0.720	Ascomycota
Wardomyces	0.448	0.516	0.006	43	0.720	Ascomycota
Mortierella	0.448	0.516	0.006	43	0.720	Mortierellomycota
Isaria	0.292	0.487	0.017	28	0.587	Ascomycota
Cephalotrichum	0.375	0.492	0.012	36	0.621	Ascomycota
Scytalidium	0.469	0.525	0.018	45	0.589	Ascomycota
Cystofilobasidium	0.469	0.525	0.018	45	0.589	Basidiomycota
unclassified_o__Auriculariales	0.229	0.466	0.015	22	0.628	Basidiomycota
Coniosporium	0.208	0.449	0.013	20	0.611	Ascomycota
unclassified_o__Glomerellales	0.313	0.503	0.041	30	0.561	Ascomycota
unclassified_o__Pleosporales	0.292	0.490	0.012	28	0.659	Ascomycota
Camarographium	0.490	0.536	0.013	47	0.635	Ascomycota
Sagenomella	0.063	0.358	0.008	6	0.667	Ascomycota
Arthrographis	0.500	0.558	0.041	48	0.561	Ascomycota
Aspergillus	0.427	0.545	0.021	41	0.630	Ascomycota
Pseudogymnoascus	0.427	0.545	0.021	41	0.630	Ascomycota
Cladosporium	0.177	0.447	0.087	17	0.684	Ascomycota
Paraconiothyrium	0.333	0.482	0.017	32	0.631	Ascomycota
unclassified_Mortierellomycota	0.354	0.500	0.033	34	0.569	Mortierellomycota
Phoma	0.125	0.432	0.006	12	0.606	Ascomycota
Didymella	0.323	0.513	0.011	31	0.626	Ascomycota
Fusicolla	0.323	0.513	0.011	31	0.626	Ascomycota
unclassified_f__Chaetomiaceae	0.344	0.513	0.009	33	0.655	Ascomycota
Paramyrothecium	0.344	0.513	0.009	33	0.655	Ascomycota
Lophotrichus	0.344	0.513	0.009	33	0.655	Ascomycota
Phaeosphaeria	0.344	0.513	0.009	33	0.655	Ascomycota

Gibellulopsis	0.292	0.485	0.010	28	0.638	Ascomycota
Auxarthron	0.292	0.490	0.012	28	0.659	Ascomycota
Setophaeosphaeria	0.292	0.490	0.012	28	0.659	Ascomycota
Trematophoma	0.292	0.490	0.012	28	0.659	Ascomycota
Amphobotrys	0.302	0.511	0.016	29	0.658	Ascomycota
Sebacina	0.135	0.429	0.067	13	0.423	Basidiomycota
Gemmina	0.135	0.455	0.025	13	0.372	Ascomycota
Thelephora	0.177	0.466	0.018	17	0.581	Basidiomycota
Bradymyces	0.188	0.436	0.009	18	0.641	Ascomycota
Tausonia	0.250	0.449	0.025	24	0.772	Basidiomycota
Neocosmospora	0.417	0.527	0.071	40	0.546	Ascomycota
Trichoderma	0.313	0.495	0.020	30	0.692	Ascomycota
Titaea	0.427	0.511	0.030	41	0.663	Ascomycota
Penicillium	0.427	0.511	0.006	41	0.735	Ascomycota
Chaetomium	0.427	0.511	0.006	41	0.735	Ascomycota
unclassified_f__Didymosphaeriaceae	0.427	0.511	0.006	41	0.735	Ascomycota
unclassified_Ascomycota	0.427	0.511	0.006	41	0.735	Ascomycota
Acaulium	0.427	0.511	0.006	41	0.735	Ascomycota
unclassified_o__Chaetothyriales	0.427	0.511	0.006	41	0.735	Ascomycota
unclassified_o__Xylariales	0.063	0.364	0.036	6	0.667	Ascomycota
unclassified_o__Helotiales	0.031	0.314	0.041	3	0.000	Ascomycota
unclassified_f__Boletaceae	0.052	0.375	0.051	5	0.300	Basidiomycota
unclassified_f__Arthopyreniaceae	0.042	0.366	0.021	4	0.333	Ascomycota
Paraphoma	0.010	0.274	0.000	1	0.000	Ascomycota
Phaeosphaeriopsis	0.042	0.293	0.020	4	0.333	Ascomycota
Microdochium	0.031	0.274	0.021	3	0.000	Ascomycota

unclassified_o__Branch06	0.031	0.303	0.005	3	0.000	Ascomycota
unclassified_f__Herpotrichiellaceae	0.031	0.344	0.000	3	1.000	Ascomycota
Cutaneotrichosporon	0.083	0.382	0.016	8	0.607	Basidiomycota
Paraboeremia	0.010	0.215	0.000	1	0.000	Ascomycota
Peziza	0.021	0.253	0.006	2	0.000	Ascomycota
Alternaria	0.010	0.239	0.000	1	0.000	Ascomycota
unclassified_c__Sordariomycetes	0.031	0.256	0.010	3	0.000	Ascomycota
unclassified_f__Mycosphaerellaceae	0.031	0.262	0.028	3	0.000	Ascomycota
Talaromyces	0.021	0.303	0.017	2	0.000	Ascomycota
Phialosimplex	0.010	0.208	0.000	1	0.000	Ascomycota
Dactylonectria	0.042	0.331	0.050	4	0.333	Ascomycota
Oidiodendron	0.021	0.230	0.000	2	1.000	Ascomycota
Cercospora	0.042	0.264	0.006	4	0.500	Ascomycota
Exophiala	0.042	0.353	0.017	4	0.333	Ascomycota
Holtermanniella	0.021	0.267	0.000	2	1.000	Basidiomycota
Sordaria	0.031	0.315	0.005	3	0.333	Ascomycota
Spiromastix	0.271	0.427	0.000	26	0.926	Ascomycota
unclassified_c__Eurotiomycetes	0.292	0.449	0.000	28	0.942	Ascomycota
Infundichalara	0.292	0.449	0.000	28	0.942	Ascomycota
unclassified_f__Hyaloscyphaceae	0.042	0.375	0.000	4	0.833	Ascomycota
Purpureocillium	0.042	0.357	0.001	4	0.667	Ascomycota
Pseudombrophila	0.083	0.398	0.012	8	0.536	Ascomycota
unclassified_c__Dothideomycetes	0.052	0.330	0.021	5	0.400	Ascomycota
unclassified_f__Nectriaceae	0.010	0.269	0.000	1	0.000	Ascomycota

To gain a deeper insight into the interaction among fungi from soil samples of *Verticillium* wilt-affected plants, the fungi in the rhizosphere

(RHS) and non-rhizosphere (NRHS) soils were merged (FPS) to construct the co-occurrence network. A connection denoted a strong (Spearman's ρ>0.8 or <-0.8) and significant (P< 0.05) correlation.

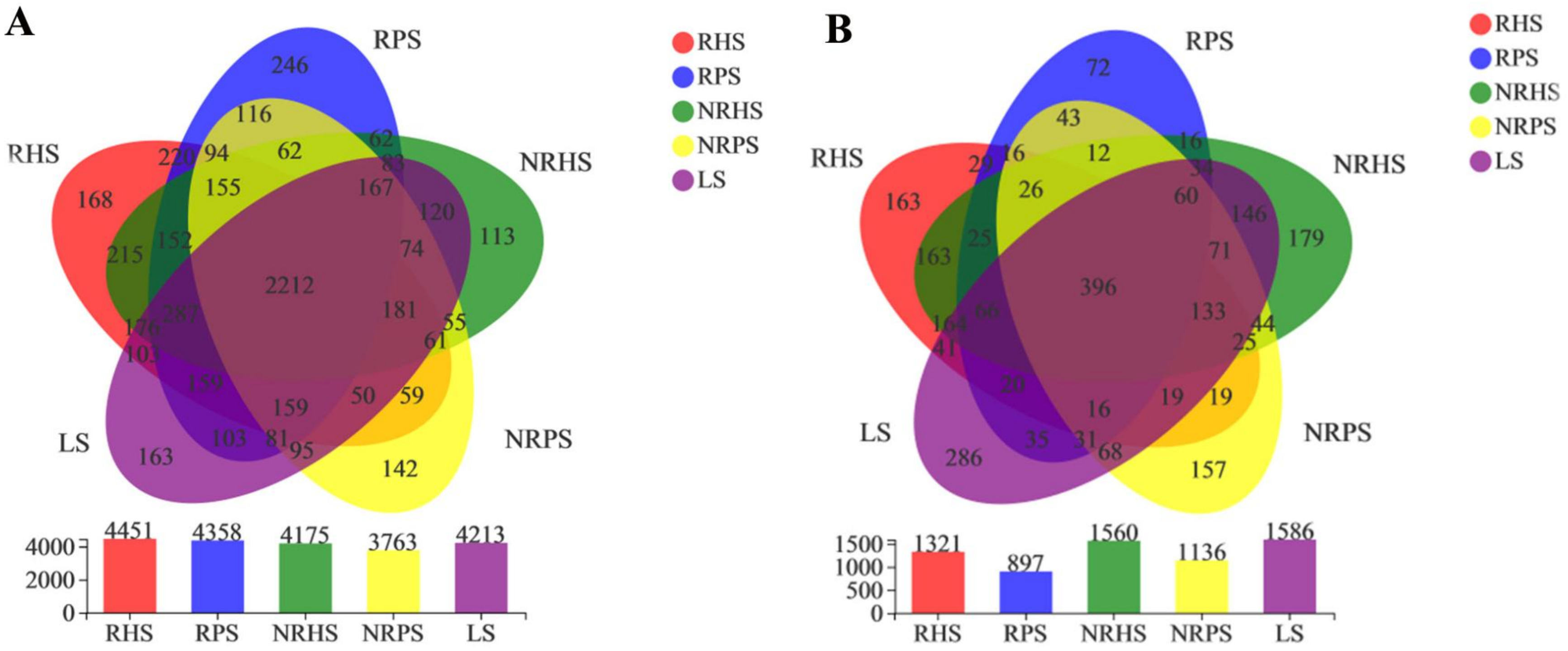

Supplementary Figure S1 Venn diagrams showing the numbers of shared and unique bacterial (A) and fungal (B) OTUs in *Cotinus coggygria* soil microbiome under different conditions. The different colored ovals represented different soil samples, and the intersections of the colored ovals indicated the coexisted bacterial or fungal OTUs in the samples. RHS: rhizosphere soil of healthy plants; RPS: rhizosphere soil of *Verticillium* wilt-affected plants; NRHS: non-rhizosphere soil of healthy plants; NRPS: non-rhizosphere soil of *Verticillium* wilt-affected plants; LS: bulk soil in the *C. coggygria* forest.

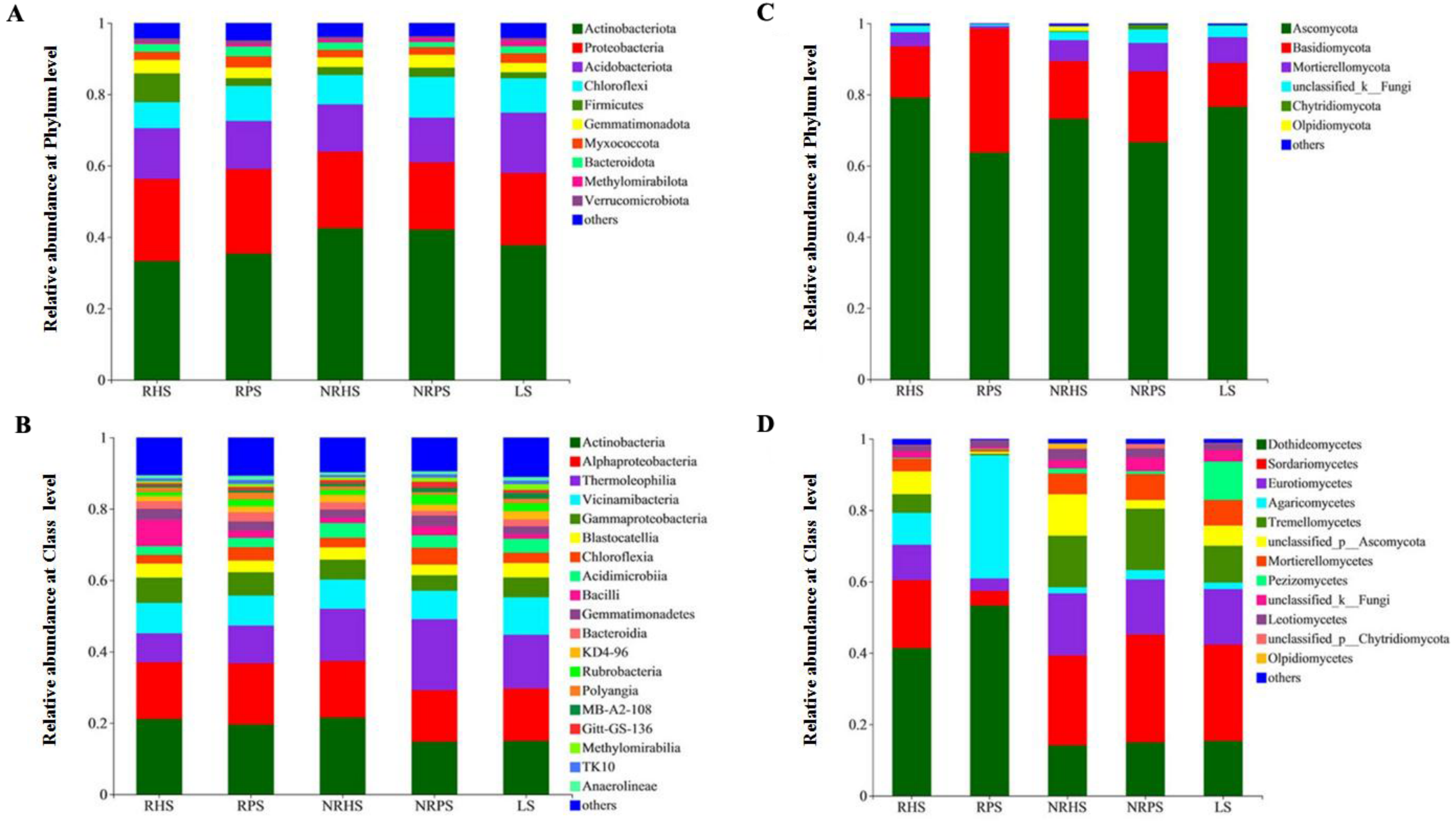
A
Relative abundance at Phylum level
RHS
RPS
NRHS
NRPS
LS
Actinobacteriota
Proteobacteria
Acidobacteriota
Chloroflexi
Firmicutes
Gemmatimonadota
Myxococcota
Bacteroidota
Methylomirabilota
Verrucomicrobiota
others
B
Relative abundance at Class level
Actinobacteria
Alphaproteobacteria
Thermoleophilia
Vicinamibacteria
Gammaproteobacteria
Blastocatellia
Chloroflexia
Acidimicrobiia
Bacilli
Gemmatimonadetes
Bacteroidia
KD4-96
Rubrobacteria
Polyangia
MB-A2-108
Gitt-GS-136
Methylomirabilia
TK10
Anaerolineae
others
C
Relative abundance at Phylum level
Ascomycota
Basidiomycota
Mortierellomycota
unclassified_k__Fungi
Chytridiomycota
Olpidiomycota
others
D
Relative abundance at Class level
Dothideomycetes
Sordariomycetes
Eurotiomycetes
Agaricomycetes
Tremellomycetes
unclassified_p__Ascomycota
Mortierellomycetes
Pezizomycetes
unclassified_k__Fungi
Leotiomycetes
unclassified_p__Chytridiomycota
Olpidiomycetes
others

Supplementary Figure S2 Community composition of *Cotinus coggygria* soil microbiome under different conditions. Bacterial community composition at phylum (A) and class (B) levels. Fungal community composition at phylum (C) and class (D) levels. Others indicated the taxa with relative abundance lower than 0.1%.

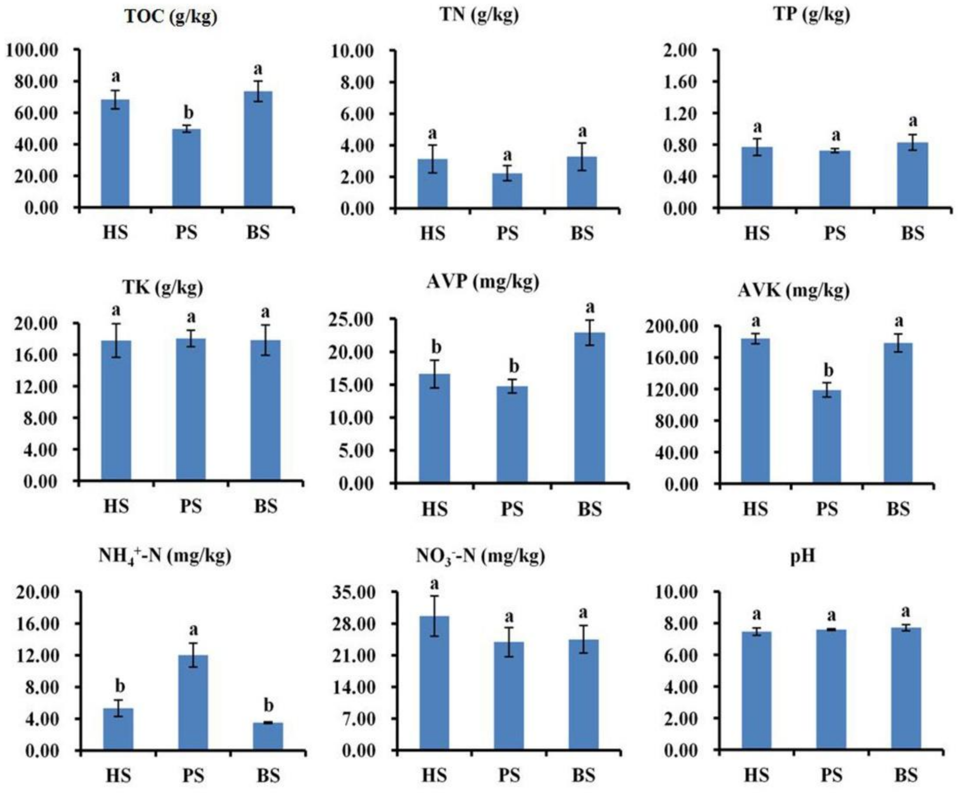

Supplementary Figure S3 Physicochemical properties of *Cotinus coggygria* soil samples under different conditions. HS: soil samples from healthy *Cotinus coggygria* plants; PS: soil samples from *Verticillium* wilt-affected plants; BS: bulk soil in the *C. coggygria* forest. TOC: total organic carbon, TN: total nitrogen, TP: total phosphorus, TK: total potassium, AVP: available phosphorus, AVK: available potassium, NH_4^+-N: ammonium nitrogen, NO_3^--N: nitrate nitrogen.

Welch's t-test bar plot at Genus level

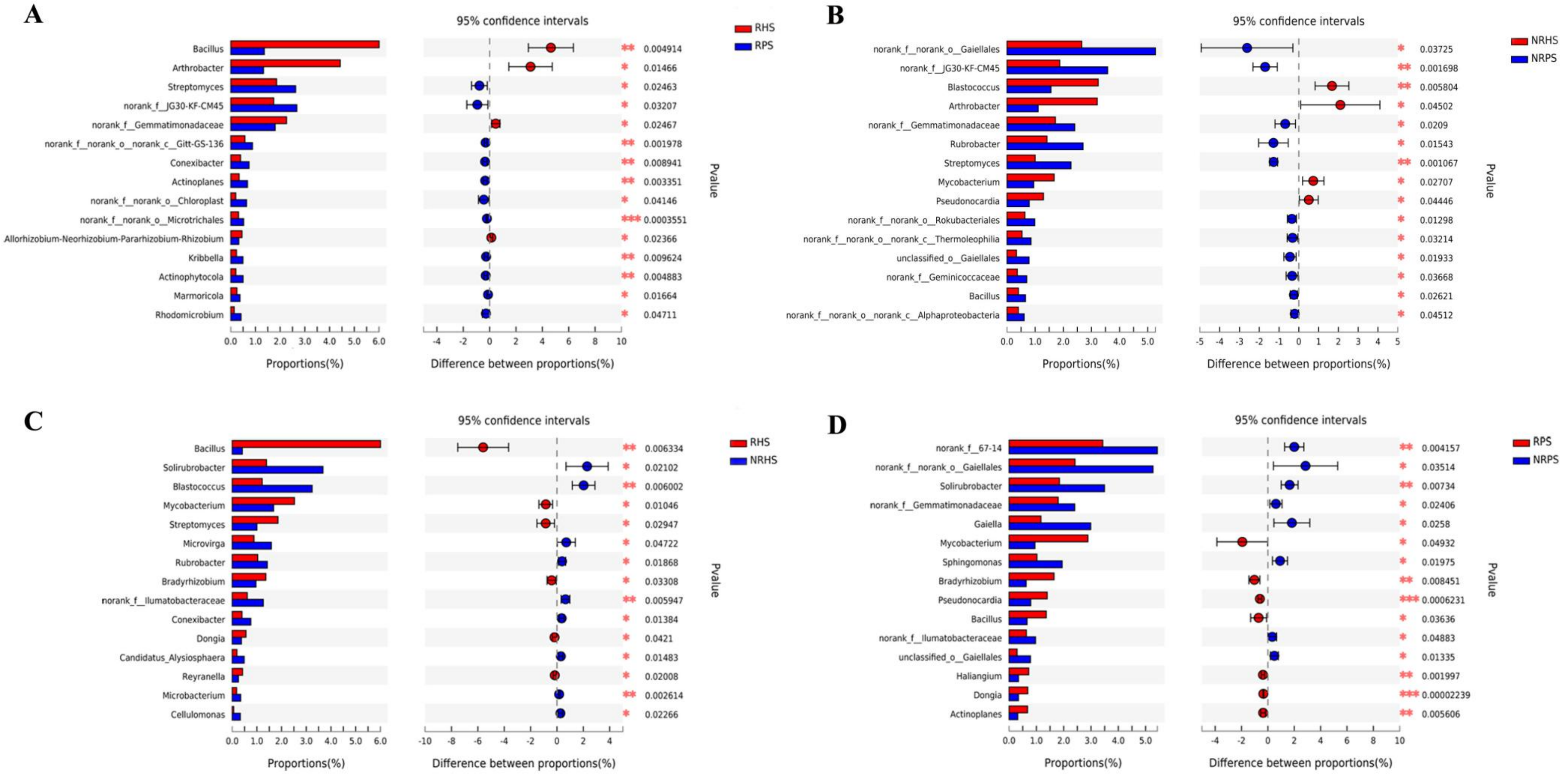

Supplementary Figure S4 Welch's t-test and FDR comparison analysis of some significantly enriched bacterial genera between samples from different plant healthy status or soil compartments. (A) RHS vs RPS, (B) NRHS vs NRPS, (C) RHS vs NRHS, (D) RPS vs NRPS. The Scheffe's value cutoff was 0.95, ***: $P \leq 0.001$, **: $0.001 < P \leq 0.01$, *: $0.01 < P \leq 0.05$. RHS: rhizosphere soil of healthy plants; RPS: rhizosphere soil of *Verticillium* wilt-affected plants; NRHS: non-rhizosphere soil of healthy plants; NRPS: non-rhizosphere soil of *Verticillium* wilt-affected plant.

Welch’s t-test bar plot at Genus level

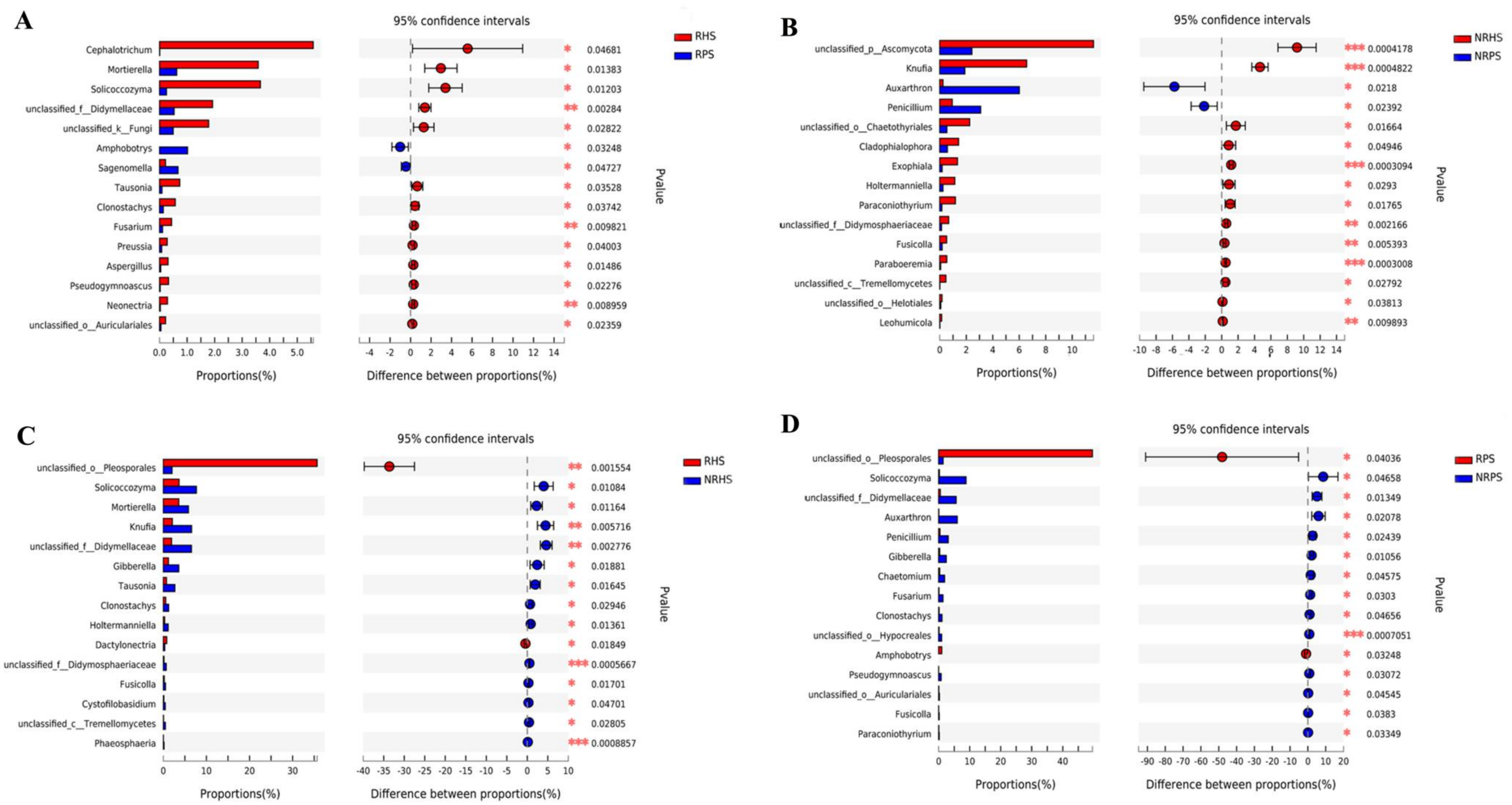

Supplementary Figure S5 Welch's t-test and FDR comparison analysis of some significantly enriched fungal genera between samples from different plant healthy status or soil compartments. (A) RHS vs RPS, (B) NRHS vs NRPS, (C) RHS vs NRHS, (D) RPS vs NRPS. The Scheffe's value cutoff was 0.95, ***: $P \leq 0.001$, **: $0.001 < P \leq 0.01$, *: $0.01 < P \leq 0.05$. RHS: rhizosphere soil of healthy plants; RPS: rhizosphere soil of *Verticillium* wilt-affected plants; NRHS: non-rhizosphere soil of healthy plants; NRPS: non-rhizosphere soil of *Verticillium* wilt-affected plant.

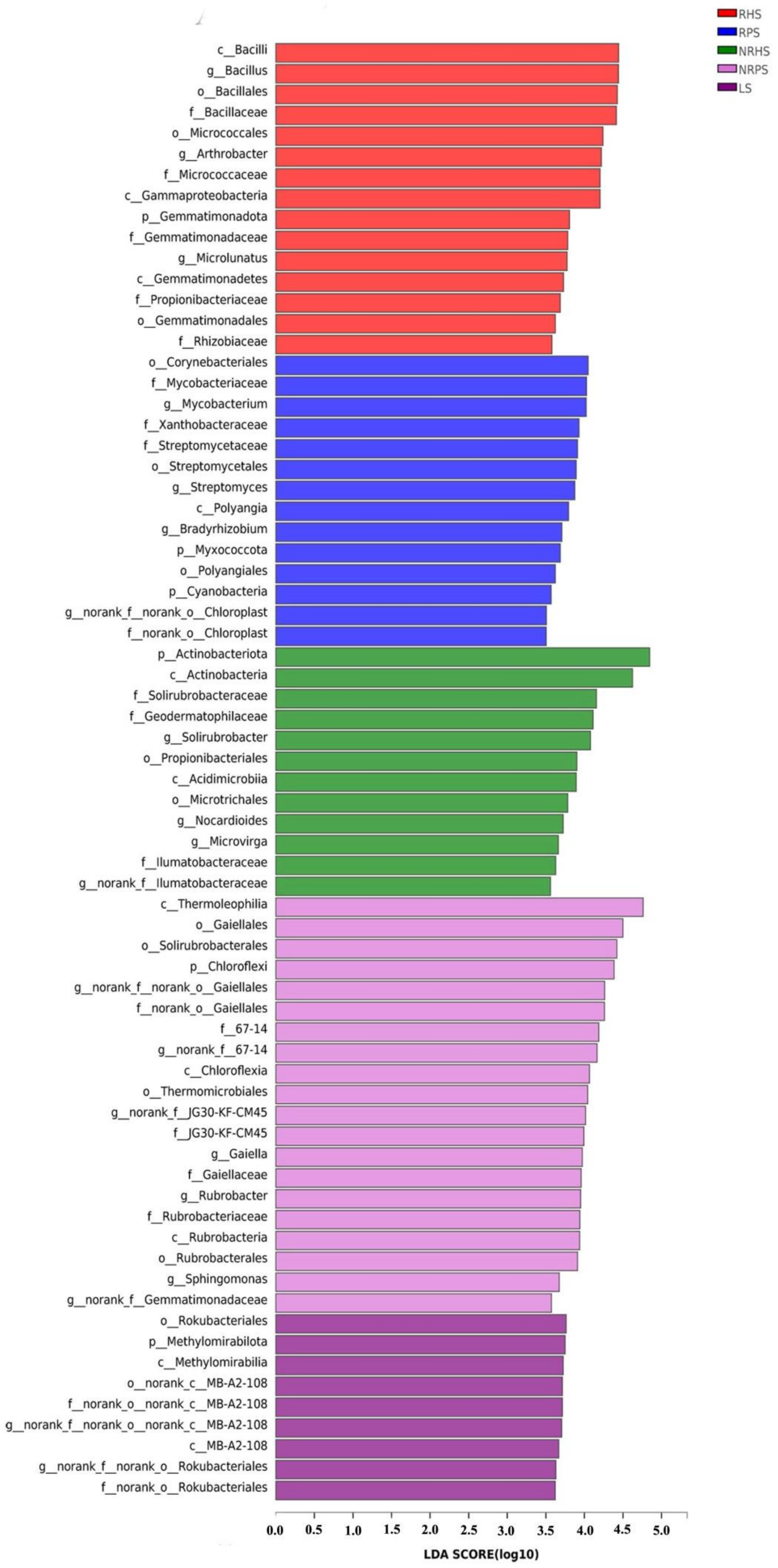
RHS
RPS
NRHS
NRPS
LS
c__Bacilli
g__Bacillus
o__Bacillales
f__Bacillaceae
o__Micrococcales
g__Arthrobacter
f__Micrococcaceae
c__Gammaproteobacteria
p__Gemmatimonadota
f__Gemmatimonadaceae
g__Microlunatus
c__Gemmatimonadetes
f__Propionibacteriaceae
o__Gemmatimonadales
f__Rhizobiaceae
o__Corynebacteriales
f__Mycobacteriaceae
g__Mycobacterium
f__Xanthobacteraceae
f__Streptomycetaceae
o__Streptomycetales
g__Streptomyces
c__Polyangia
g__Bradyrhizobium
p__Myxococcota
o__Polyangiales
p__Cyanobacteria
g__norank_f__norank_o__Chloroplast
f__norank_o__Chloroplast
p__Actinobacteriota
c__Actinobacteria
f__Solirubrobacteraceae
f__Geodermatophilaceae
g__Solirubrobacter
o__Propionibacteriales
c__Acidimicrobiia
o__Microtrichales
g__Nocardioides
g__Microvirga
f__Ilumatobacteraceae
g__norank_f__Ilumatobacteraceae
c__Thermoleophilia
o__Gaiellales
o__Solirubrobacterales
p__Chloroflexi
g__norank_f__norank_o__Gaiellales
f__norank_o__Gaiellales
f__67-14
g__norank_f__67-14
c__Chloroflexia
o__Thermomicrobiales
g__norank_f__JG30-KF-CM45
f__JG30-KF-CM45
g__Gaiella
f__Gaiellaceae
g__Rubrobacter
f__Rubrobacteriaceae
c__Rubrobacteria
o__Rubrobacterales
g__Sphingomonas
g__norank_f__Gemmatimonadaceae
o__Rokubacteriales
p__Methylomirabilota
c__Methylomirabilia
o__norank_c__MB-A2-108
f__norank_o__norank_c__MB-A2-108
g__norank_f__norank_o__norank_c__MB-A2-108
c__MB-A2-108
g__norank_f__norank_o__Rokubacteriales
f__norank_o__Rokubacteriales
0.0 0.5 1.0 1.5 2.0 2.5 3.0 3.5 4.0 4.5 5.0
LDA SCORE(log10)

Supplementary Figure S6 Histogram of the LDA scores showing crucial bacterial biomarkers (from phylum to genus levels) in *Cotinus coggygria* soil microbiome under different conditions. Significant differences were defined at $P < 0.05$ with an LDA score > 3.5.

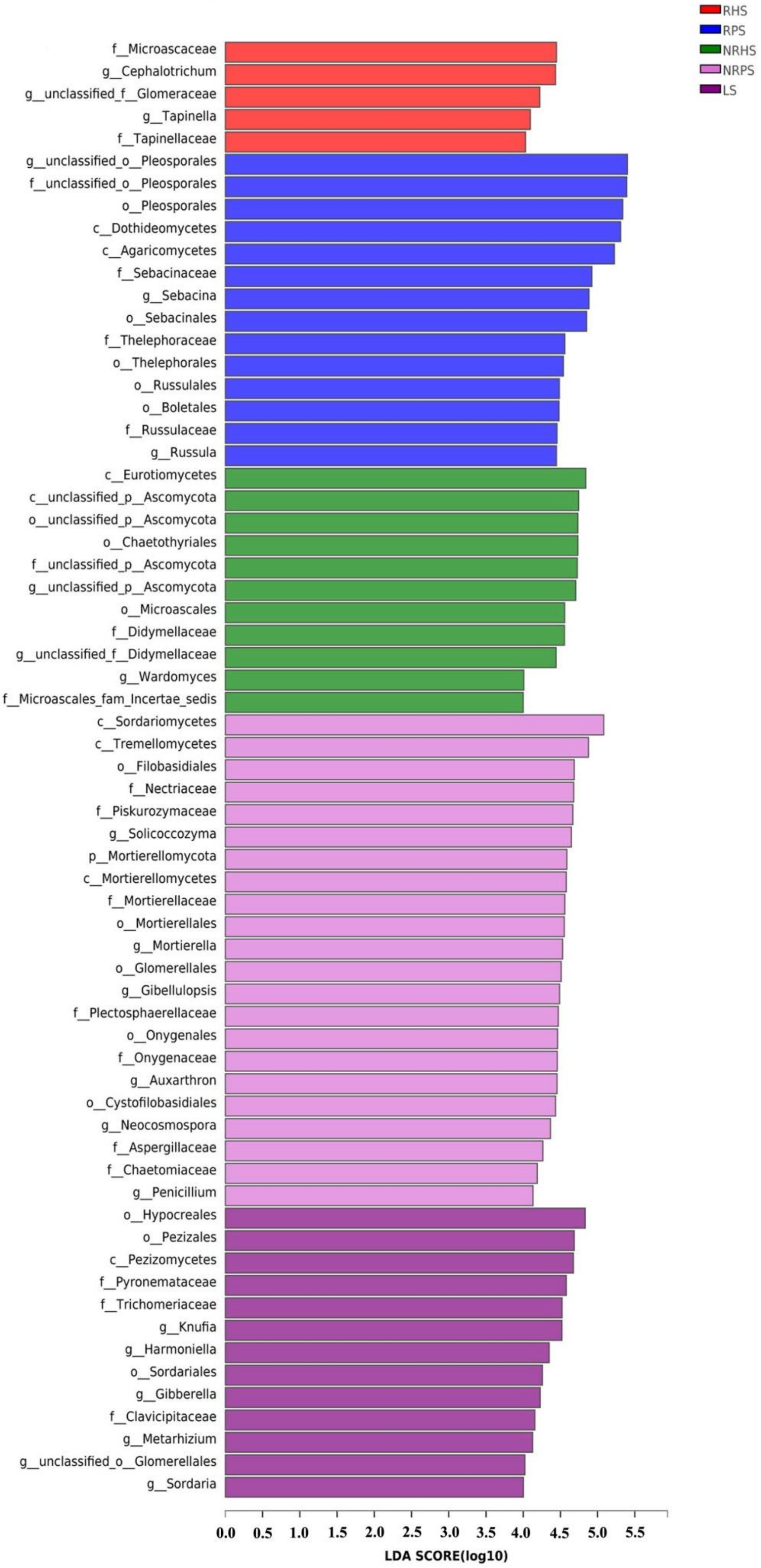

RHS
RPS
NRHS
NRPS
LS
f__Microascaceae
g__Cephalotrichum
g__unclassified_f__Glomeraceae
g__Tapinella
f__Tapinellaceae
g__unclassified_o__Pleosporales
f__unclassified_o__Pleosporales
o__Pleosporales
c__Dothideomycetes
c__Agaricomycetes
f__Sebacinaceae
g__Sebacina
o__Sebacinales
f__Thelephoraceae
o__Thelephorales
o__Russulales
o__Boletales
f__Russulaceae
g__Russula
c__Eurotiomycetes
c__unclassified_p__Ascomycota
o__unclassified_p__Ascomycota
o__Chaetothyriales
f__unclassified_p__Ascomycota
g__unclassified_p__Ascomycota
o__Microascales
f__Didymellaceae
g__unclassified_f__Didymellaceae
g__Wardomyces
f__Microascales_fam_Incertae_sedis
c__Sordariomycetes
c__Tremellomycetes
o__Filobasidiales
f__Nectriaceae
f__Piskurozymaceae
g__Solicoccozyma
p__Mortierellomycota
c__Mortierellomycetes
f__Mortierellaceae
o__Mortierellales
g__Mortierella
o__Glomerellales
g__Gibellulopsis
f__Plectosphaerellaceae
o__Onygenales
f__Onygenaceae
g__Auxarthron
o__Cystofilobasidiales
g__Neocosmospora
f__Aspergillaceae
f__Chaetomiaceae
g__Penicillium
o__Hypocreales
o__Pezizales
c__Pezizomycetes
f__Pyronemataceae
f__Trichomeriaceae
g__Knufia
g__Harmoniella
o__Sordariales
g__Gibberella
f__Clavicipitaceae
g__Metarhizium
g__unclassified_o__Glomerellales
g__Sordaria
0.0 0.5 1.0 1.5 2.0 2.5 3.0 3.5 4.0 4.5 5.0 5.5
LDA SCORE(log10)

Supplementary Figure S7 Histogram of the LDA scores showing crucial fungal biomarkers (from phylum to genus levels) in *Cotinus coggygria* soil microbiome under different conditions. Significant differences were defined at $P < 0.05$ with an LDA score > 4.0.

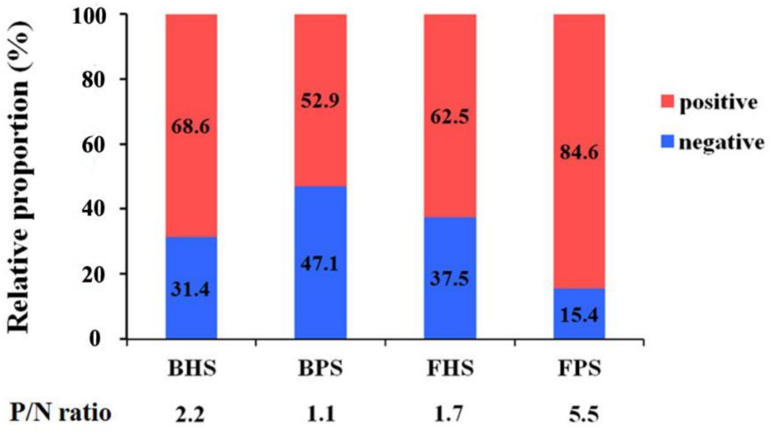

Supplementary Figure S8 Relative proportion of positive and negative interactions in microbial co-occurrence networks of healthy and *Verticillium* wilt-affected *Cotinus coggygria*. The ratios of positive to negative interactions in different samples were displayed as P/N ratio in the below of the bar chart. BHS: network of soil bacteria from healthy plants; BPS: network of soil bacteria from *Verticillium* wilt-affected plants; FHS: network of soil fungi from healthy plant; FPS: network of soil fungi from *Verticillium* wilt-affected plants.

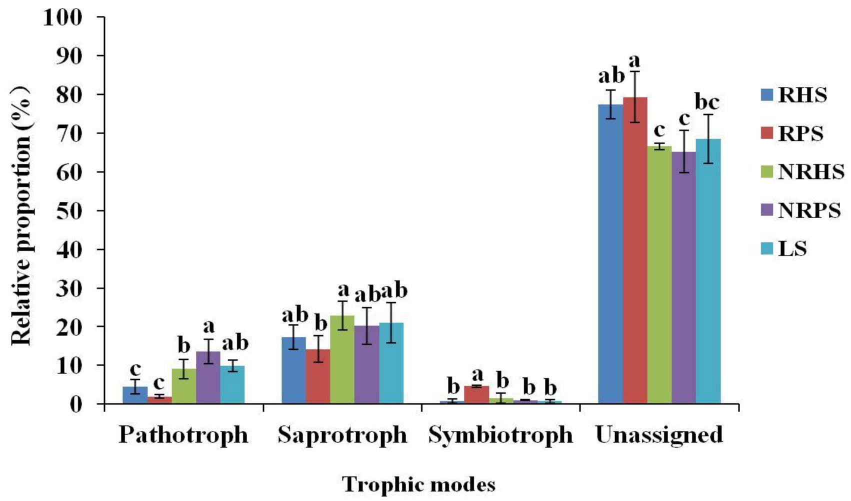

Supplementary Figure S9 Relative proportion of fungal trophic modes in *Cotinus coggygria* soil microbiome under different conditions based on FUNGuild database. RHS: rhizosphere soil of healthy plants; RPS: rhizosphere soil of *Verticillium* wilt-affected plants; NRHS: non-rhizosphere soil of healthy plants; NRPS: non-rhizosphere soil of *Verticillium* wilt-affected plants; LS: bulk soil in the *C. coggygria* forest.
